# Adsorption characteristics and applications of andesite in removing some pollutants from wastewater

**DOI:** 10.1038/s41598-024-65043-y

**Published:** 2024-07-05

**Authors:** Abdalla M. Khedr, Nadia Elwakiel, Sameh E. Halawia, Ramadan Abdelghany Mansour

**Affiliations:** 1 Chemistry Department, Faculty of Science, Tanta University, Tanta, 31527 Egypt; 2 Basic Sciences and Engineering Department, Higher Institute of Engineering and Technology, New Damietta, 34517 Egypt

**Keywords:** Andesite, Adsorption, Desorption, Langmuir isotherm, Freundlich, Environmental sciences, Chemistry

## Abstract

Andesite was employed to effectively extract mercury(II) in an aqueous solution. After evaluating its characteristics, andesite was characterized by applying modern techniques such as BET and TGA methods. The study employed SEM and TEM measurements to analyze the variation in the surface shape and crystallinity of the metal due to adsorption. Using the EDX process, the chemical composition, weight, and atomic percentage of each element of andesite were determined. FTIR techniques were also used to confirm the TEM–EDX findings. Zeta potential was estimated. Cycles of regeneration and desorption have been examined. 99.03% was the highest uptake percentage. Adsorbent quantity (0.0025–0.05) g/L, contact time (5–60) min, pH (2–10), temperature (25–60) °C, and dose (0.0027, 0.0044, 0.0125, 0.0155, and 0.0399) mg/L all affect the amount of removal that increases with the increase in contact time, pH, dose, and temperature but drops as the metal ion concentration rises. The ideal values for contact time, pH, metal ion concentration, dose, and temperature were found to be, respectively, 30 min, 0.0155 mg/l, 0.02 g/l, and 40 °C. The calculation of thermodynamic parameters, including ΔH, ΔG, and ΔS, was imperative in establishing that the mechanism of heavy metal adsorption on andesite was endothermic, exhibiting a physical nature that escalated with temperature rise. The Freundlich adsorption equation's linear form is matched by the adsorption of mercury(II) on andesite; constant n was 1.85, 1.06, 1.1, and 1.1, whereas the Langmuir constant q_m_ was found to be 1.85, 2.41, 3.54, and 2.28 mg/g at 25–60 °C. Furthermore, adsorption follows a pseudo-second-order rate constant of (3.08, 3.24, 3.24, and 13) g/mg/min under identical temperature conditions, as opposed to a first-order rate constant of 4, 3, 2.6, and 2. Hg^2+^, NH_4_^+^, Cl^−^, Br^−^, NO_3_^−^, SO_4_^2−^, Na^+^, K^+^, H_2_S, and CH_3_SH were all extracted from wastewater by this application.

## Introduction

Mercury(II) can find its way into the environment and water supplies either naturally, through processes like mineral deposit erosion and volcanic activity, or artificially, through mining, metallurgy, coal production, coal-fired power plants, residential heating systems, paper pulp manufacturing, incinerators, and chemical synthesis^1^. Fish are the first to ingest methylmercury, which is produced when mercury molecules in the environment change. Following its distribution from the gastrointestinal tract to the kidneys and central nervous system in humans, methylmercury causes haematological problems, cardiovascular ailments^2,3^, seizures, cerebral palsy, and other conditions. The World Health Organization's (WHO) recommended standards are shown in Table 1^4^. Mercury(II) has been removed through a variety of techniques, including biological^5^, coagulation-flocculation^6^, membrane technology^7^, and adsorption^8,9^ procedures. The environmental friendliness, inexpensive cost, high extraction level, simple design, ease of functioning, comparatively minimal sludge output, and adsorbent renewal capacity^10–13^ make adsorption procedures appealing.

**Table 1:** World Health Organization (WHO) standard for heavy metals.

S/N	Metal	Highest desirable (mg/L)	Max. desirable (mg/L)
1	Fe	1.0	3.0
2	Cu	0.5	2.0
3	Zn	1.0	3.0
4	Pb	0.4	0.4
5	Ni	0.01	0.02
6	Cr	0.05	0.05
7	Cd	0.003	0.03
8	As	0.01	0.01
9	Ba	0.05	0.05
10	Hg	0.001	0.001
11	Sb	0.01	0.02
12	Sn	0.01	1.0
13	Se	0.01	0.01
14	Mn	0.4	0.4

The suggested method’s performance was compared to various adsorbents. Table 2 compares the highest mercury(II) ion adsorption efficiency utilizing various adsorbent materials that have been used for mercury(II) adsorption in the past.

**Table 2:** Hg(II) maximum adsorption capacity comparison using various adsorbents.

Adsorbents	Adsorbent efficiency (mg/g)
Dsss^13^	35.14
Blp^14^	27.1
Riton x-100 modified blp^14^	28.1
Tsds modified blp^14^	31.05
Yarrowia lipolytica 70562^15^	181.83
Charcoal-immobilized Papain (cip)^16^	4.70
Camel bone charcoal^17^	28.24
Lemna minor powder^18^	27.62
Coconut shell^19^	15.19
Eucalyptus bark^20^	33.1
Expanded perlite^21^	8.46
Ethylene diamine modified^22^	30.78
Andesite^Present work^	3.54

Conventional adsorbents with a single mode of adsorption, including bentonite, goethite, and marine macroalga, have poor removal efficiencies and limited adsorption capacities for mercury (II).Thus, scientists are still looking for novel, effective adsorbents. Based on hierarchical structure, the current technique for Hg(II) removal combines the benefits of chemical and physical adsorption. Numerous Hg(II) adsorbents have been investigated to date. Adsorbents that have recently been produced comprise silica, hydroxyapatite, activated carbon, multiwalled carbon nanotubes (MWCNTs), two-dimensional metal carbides, several polymer types, etc^23^. Hg(II) removal employing green-prepared reduced graphene oxide (rGO), which can successfully lower the Hg(II) concentration of 150 mg/L at a low level of less than 40 mg/L and demonstrates an intriguing Hg(II) saturation of 110.21 mg/g (at 298 K). Moreover, rGO outperforms standard Hg(II) removal adsorbents^24^ in 20 min, effectively removing over 75% Hg(II). Equipped with Rhodobacter sphaeroides SC01^25^, mercury(II) was eliminated.

**Figure 1:**
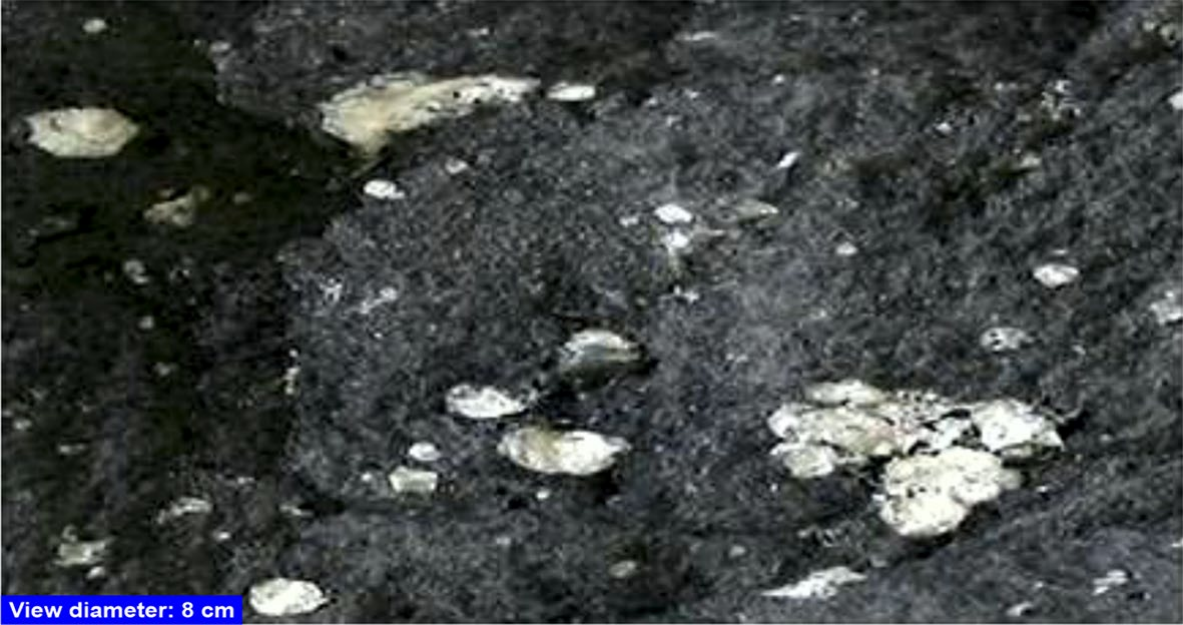
A sample of andesite (dark groundmass) with zeolite-filled amygdaloidal vesicles.

Andesite is a volcanic rock that extrudes and has an intermediate composition^26^. It is generally thought of as the kind in between rhyolite and basalt. Its texture ranges from fine-grained (aphanitic) to porphyritic, and its main constituents are plagioclase, which is rich in sodium, with either pyroxene or hornblende^27^ (Fig. 1). The Andes Mountain range, which has an abundance of this type of rock, is the source of the name andesite. Andesite is an aphanitic igneous rock with fine grains that has an intermediate silica content and a small amount of alkali metals. Plagioclase^28^ contains at least 65% of the feldspar in the rock, with less than 20% of the volume being quartz and 10% being feldspathoid. Chemically speaking, andesite is described as a volcanic rock that contains between 57 and 63% silica and no more than 6% alkali metal oxides^29^.

The composition of andesite ranges from 50 to 56% by volume, which is an optimum proportion of silica among that of basalt (SiO_2_ < 53%) and rhyolite (SiO_2_ > 69%)^30^.

Andesite, also marketed as wheat-rice-stone, has gained popularity due to advertising campaigns in the media in the last few years. These advertisements suggest that andesite may have some cation-exchange capacity (CEC), which could be useful for health and environmental protection programs like odor and taste absorption and drinking water softening and purification. This natural mineral, which was distinguished by its unusually large light-colored feldspar phenocrysts buried in the dark-colored groundmass, is widely distributed in the Coastal Range of Eastern Taiwan. It was also discovered that the clay mineral was rich in the presence of the expandable vermiculite, or highly charged smectite, which is expected to be Fe-rich saponite, by looking at its mineralogical compositions through X-ray diffraction (XRD). Andesite's cation-exchangeable and 2:1-form aluminosilicate features in the smectite/saponite family of clay minerals make it a potent and prospective adsorbent for the adsorption or removal of metal and organic cations from aqueous solutions.

The analysis of N_2_ adsorption–desorption in the researchers' laboratory, fortunately, indicated that the clay sample's specific surface area was preliminary and that its BET surface area was less than 5 m^2^/g^31^. Several studies used andesite as an adsorbent, and weathered basaltic andesite products (WBAP) as a potential sorbent were assessed for the removal of Ni (II) from electroplating industrial wastewater^32^ Volatile organic acid was adsorbed by porphyritic andesite^33^; it has the ability to be activated and used for cationic dye removal^31^, and zeolite was produced by the alkali activation of andesite rock and then used for dye removal^34^. The study aims to evaluate the utilization of andesite as an inexpensive adsorbent based on the above andesite adsorption performance and its relationship to certain contaminants found in wastewater, such as Hg(II), cations, and gases, as well as their adverse impacts on public health. It carefully examines how several factors, such as contact time, pH, adsorbent dosage, initial concentration, temperature, thermodynamics, kinetics, and isotherm studies, affect the removal of Hg(II) from the andesite. Furthermore, andesite has been employed as an adsorbent to remove specific gases and cations from real wastewater samples due to its large surface area and porosity. This suggests that andesite could prove to be a useful adsorbent in the near future, with the potential to impact large-scale applications and wastewater treatment technology.

## Experimental section

### Materials and methods

#### Adsorbent

Because andesite is a natural stone, it was supplied by Egypt. After a 12-h soak, it was cleaned with deionized water. After being dried for 24 h at 60 °C, it was sieved through a 200 µm mesh screen and stored in a glass jar.

#### Adsorbate

For each metal, specifically Hg(II), certified reference material (CRM) was extracted from inorganic ventures at a concentration of 1000 ± 3 µg/ml or 1000 mg/L. This allowed for the preparation of the desired concentration for the target to investigate various conditions and ultimately obtain the ideal conditions. Additionally, a quality control standard was injected into each batch to verify the validity of the findings. The target adsorbate concentrations are (0.0027, 0.0044, 0.0125, 0.0155, and 0.0399) mg/L in order. An ultrasonic instrument was utilized to eliminate any potential gases once the standards were prepared.

#### Adsorption process

**Figure 2:**
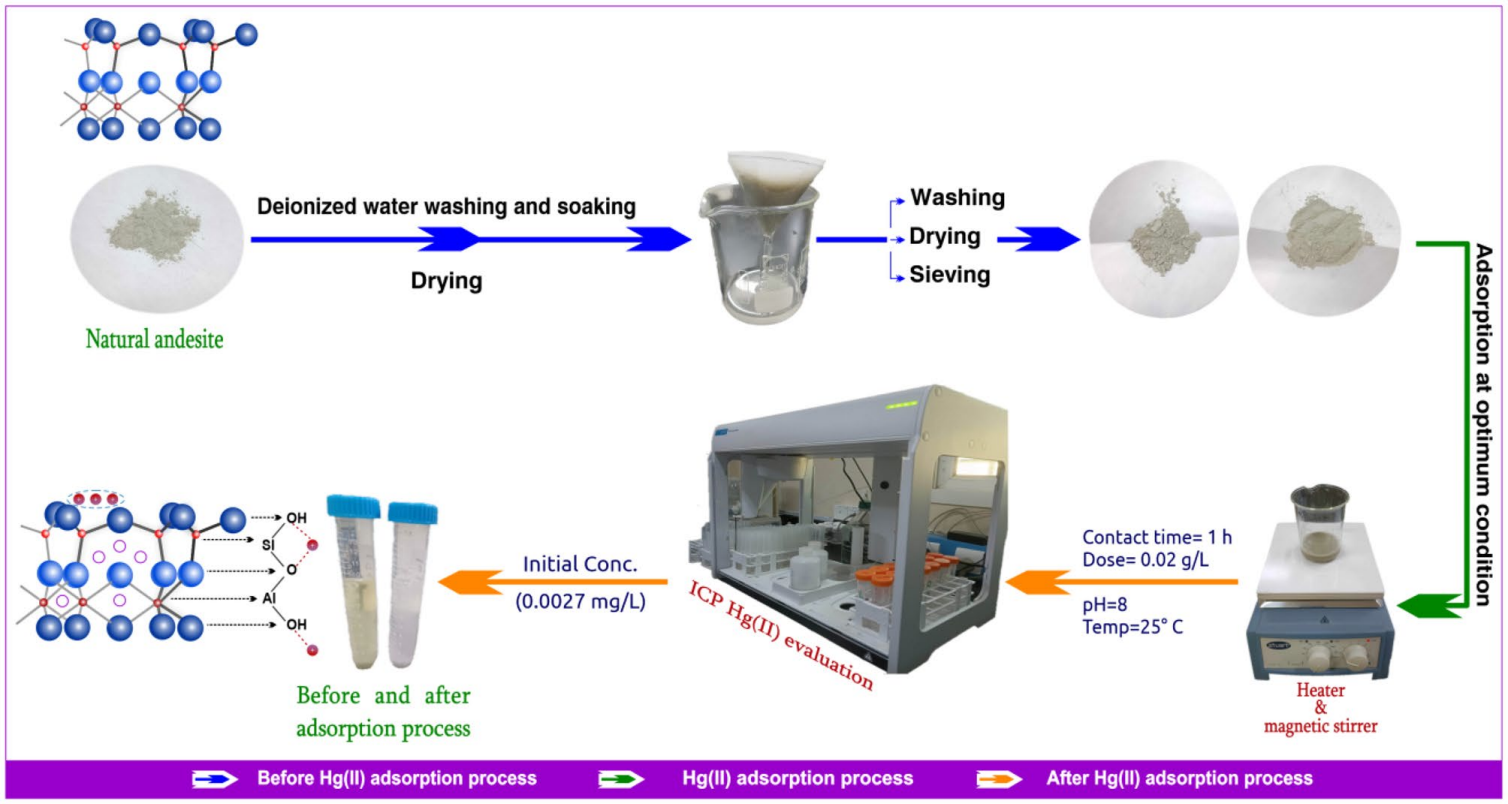
Representative diagram of mercury(II) adsorption process.

All adsorption technique tests were conducted using capped 50 mL borosilicate or polyethylene bottles with 0.02 g/L of adsorbent (andesite) and 20 mL of metal ion solution at optimum temperature (40 °C). Following an agitation of the suspensions, a 10-min, 2500 rpm centrifugation was performed. In order to assess the concentration of heavy metals, the filtrate or supernatant was removed and subjected to analysis using inductively coupled plasma (ICP-MS7900) that used argon and helium gases to achieve the desired temperature to change the metal’s status from excited state to ionization state. Numerous factors, including temperature, adsorbent quantity, metal ion concentration, pH, and contact time, that impact the adsorption procedure were investigated. The process’s efficiency was determined using the following formulas:

1 $$\% {\text{R}} = \left[ {{1} - \left( {{\text{C}}_{{\text{e}}} /{\text{C}}_{{\text{o}}} } \right)} \right] \times {1}00$$

2 $${\text{Q}}_{{\text{e}}} = \left( {{\text{C}}_{{\text{o}}} - {\text{C}}_{{\text{e}}} } \right){\text{V}}/{\text{W}}$$

where “C_o_” is the initial concentration of Hg(II) solution in (mg/L); “C_e_” is the final concentration of Hg(II) solution in (mg/L); ”V” is the volume of Hg(II) solution in (L); and “W” is the mass of the adsorbent in (g). “Q_e_” is the adsorption capacity at equilibrium (mg/g)^35^. The process has been explained briefly in Fig. 2.

#### Desorption study

This work's primary objective is to cut costs and reuse the adsorbent once more. The hydroxylation capacity, ionic potential, electronegativity, softness, and location all affect clay desorption, according to the Irving-Williams series. A 50-ml borosilicate bottle was used, and 20 mL (2 M) HCl was added to 0.02 g/L of andesite that was fully loaded with 0.1 mg/L of meta Hg(II). The bottles were then stirred at 150 rpm to reach the necessary temperature of 25 °C using an immersed water bath. The mercury concentration was measured using inductively coupled plasma (7900) in order to achieve equilibrium^36^.

#### Instruments

Using a Thermo Scientific-Nicolet iS10 FTIR Spectrometer (USA), Fourier transform infrared (FTIR) spectra of andesite were obtained in the 4000–400 cm^−1^ range both before and after adsorption. Using a scanning electron microscope (SEM, JEOL JSM 6510 Iv, England), the surface morphology of andesite was observed both before and after Hg(II) was adsorbed. The device used to determine Brunauer–Emmett–Teller analysis (BET) was the BELSORP-miniX-10185, Chromatic Tech, Japan. Utilizing TG-50 shimatzu, thermogravimetric analysis (TGA) was carried out. Using EDX (X-Max 20, Oxford), the chemical composition, weight, and atomic percentage of each element in andesite were measured. Gas concentration was calculated using MULTIRAE-LITE, and zeta potential was detected using Malvern-MAL1071664. Utilizing inductively coupled plasma mass spectrometry (ICP-MS7900, Agilent, Japan), the amounts of Hg(II) were ascertained. With an oven (JSR-Json-050, 111226-42, Korea), andesite was dried. All of the adsorption tests used a magnetic stirrer (Stuart, CB162, UK) and an ultrasonic (Branonic, 3510E-DTH, Mexico). The pH meter (HACH, HQ440d Multi, USA) was used to set the pH values of the solutions.

### Results and discussion

#### Characterization

Because it is essential to the adsorbent’s characteristics, andesite was identified using FTIR spectra and TGA, as well as BET and surface area measurements. The mineral's altered surface morphology and crystallinity upon sorption were examined using SEM and transmission electron microscopy (TEM).

#### Energy dispersive X-ray spectroscopy (EDX)

**Figure 3:**
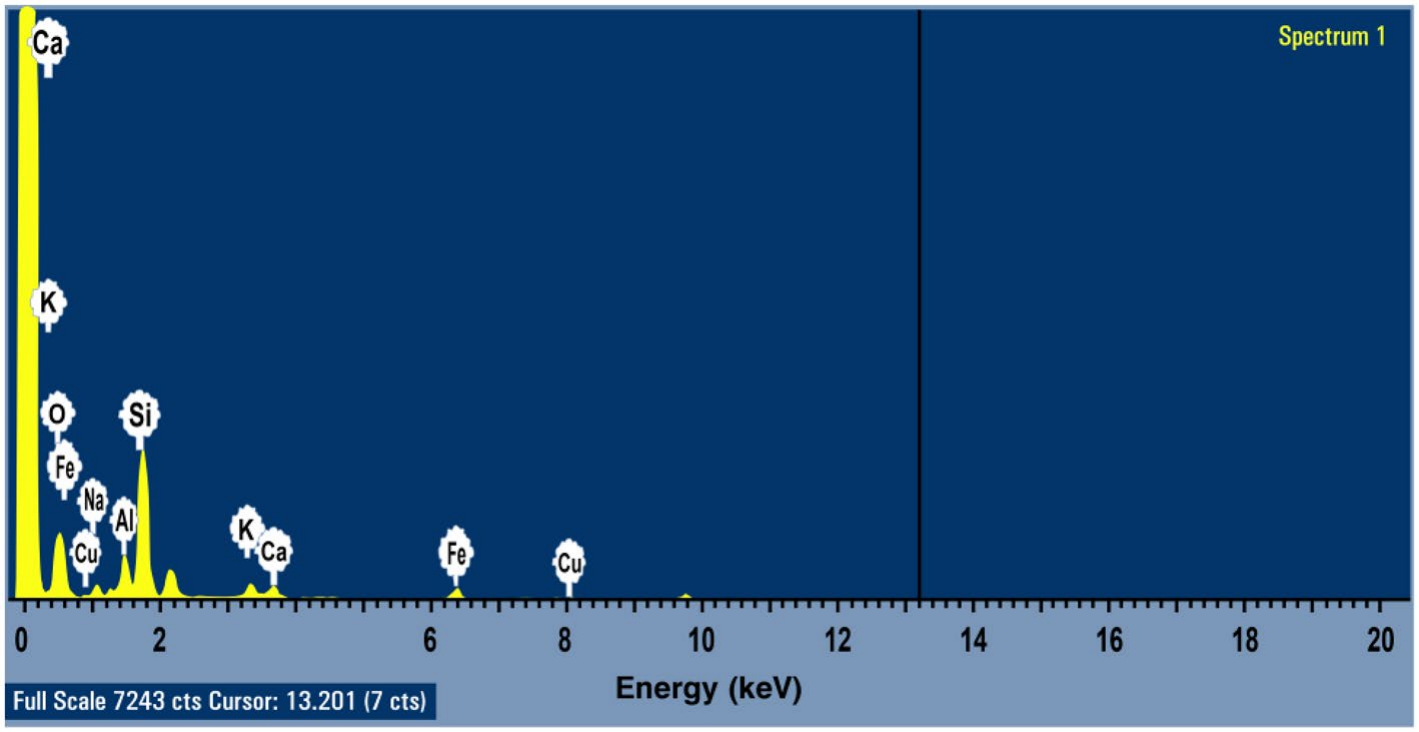
EDX characterization of andesite’s elements (O, Si, Al, Fe, Na, K, Cu, and Ca) at cursor 13.201.

**Figure 4:**
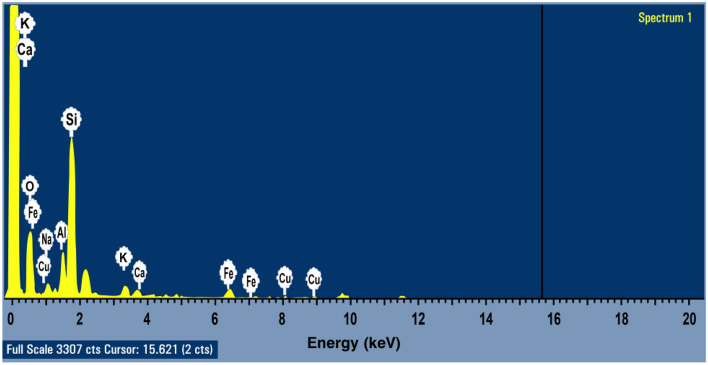
EDX characterization of andesite’s elements (O, Si, Al, Fe, Na, K, Cu, and Ca) at cursor 15.621.

The chemical composition, weight, and atomic percentage of each element in andesite, as determined by the Oxford-X-Max 20 (EDX) instrument, are displayed in Figs. 3, 4, and Table 3.

**Table 3:** Chemical composition (atomic and weight percentage of each element) of andesite.

Element	O K	Na K	Al K	Si K	K K	Ca K	Fe K	Cu K
Weight%	48.28	3.03	7.20	29.75	2.25	1.83	5.15	2.25
Atomic%	64.02	2.79	5.66	22.47	1.37	0.97	1.96	0.75

#### Fourier transform infrared (FTIR)

**Figure 5:**
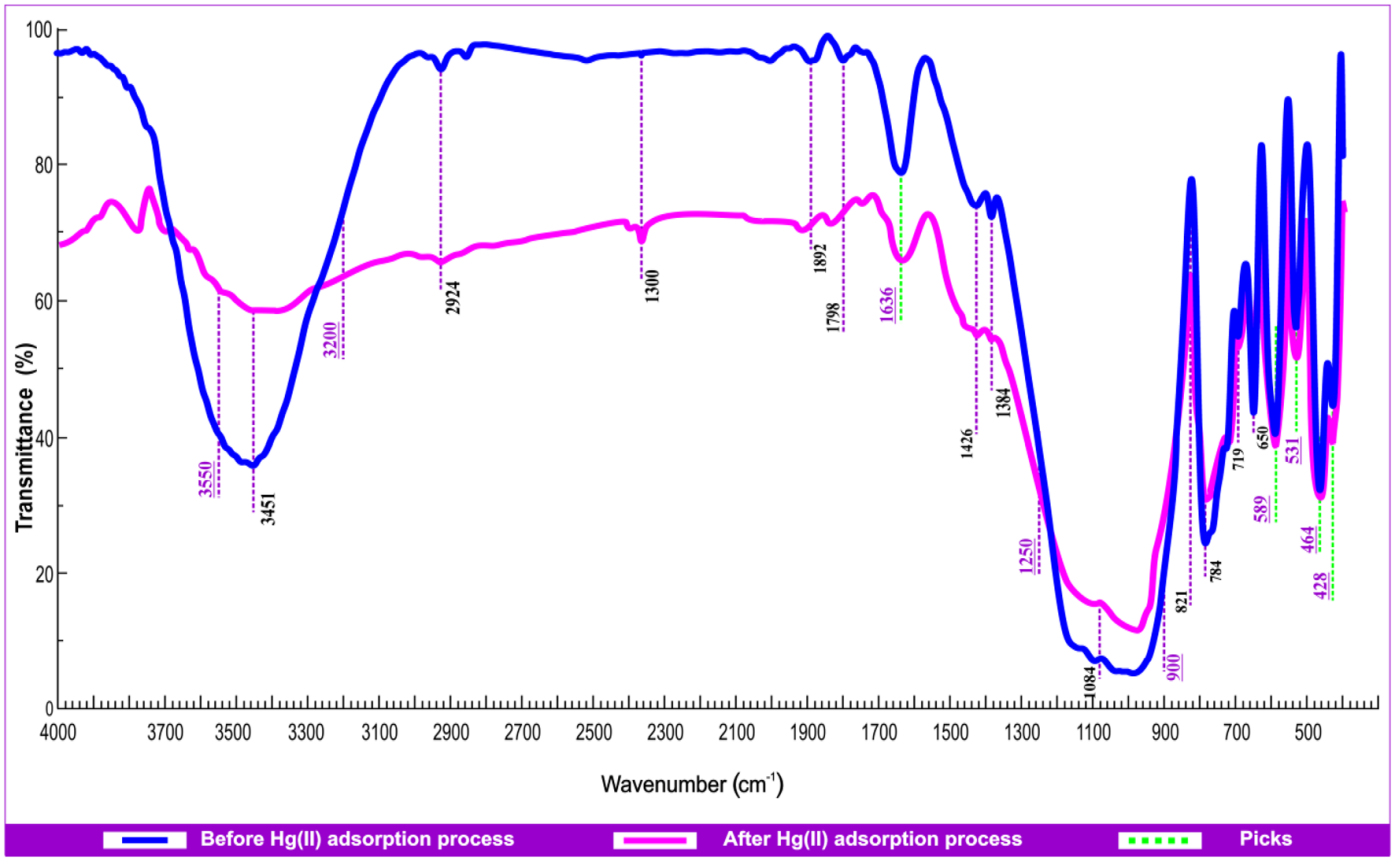
FTIR spectrophotometer analysis of andesite before and after Hg(II) adsorption.

Using FTIR (Thermo Scientific-Nicolet iS10 Spectrometer), it was found that many peaks appear, some of them broad and others weak. By spectra analysis, it was discovered that the stretching frequency in the region 3200–3550 cm^−1^ indicates the existence of a free OH group and represents the fundamental stretching vibrations of different OH^−^ groups present in Al–OH–Al and Fe–OH–Al units in the octahedral layer of the adsorbent, and the absorption peak at 1636 cm^−1^ is caused by reversibly adsorbed carbonate on the oxide surface. The steep peak that appears at 531 cm^−1^ is caused by Si–O–Al (octahedral) stretching vibrations, which are responsible for the stretching frequency in the region 900–1250 cm^−1^. The presence of calcium silicate may be the cause of the strong band at 464 cm^−1^. The peak that can be seen at 428 cm^−1^ could be an example of the asymmetric bending mode of Si–O. The existence of Al_2_O_3_ may be the cause of the peak at 589 cm^−1^. Figure 5 presents an earlier illustration of andesite before and after the Hg(II) adsorption process.

#### Brunauer–Emmett–Teller (BET)

**Figure 6:**
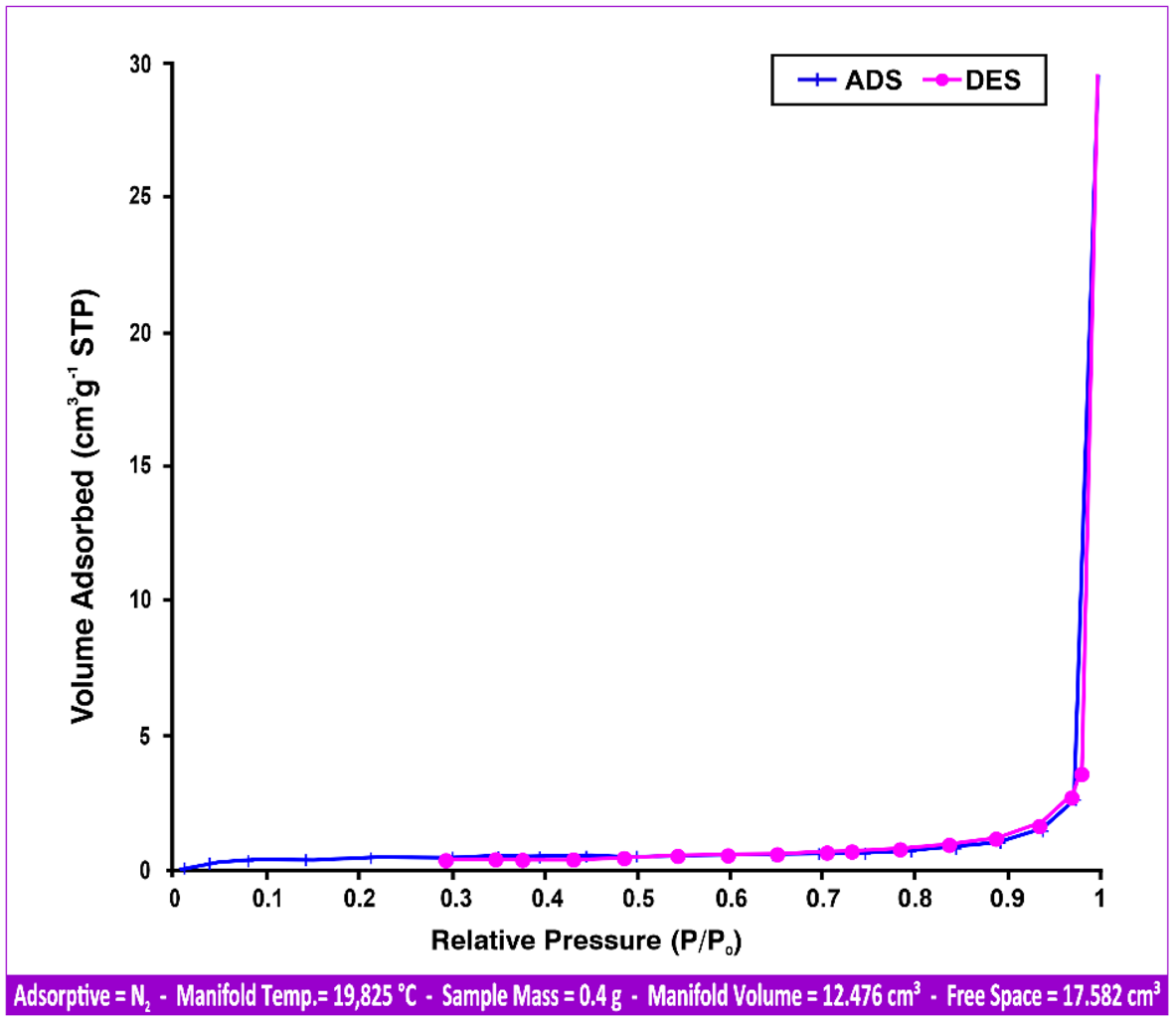
Andesite adsorption (ADS) and desorption (DES) isotherms of BET.

For andesite at 77.35 K, particular surface area and pore diameter analyses were carried out. The adsorption desorption isotherm, as can be seen in Fig. 6, was determined as a type III isotherm, or an isotherm of macroporous solid materials^37^. The average pore diameter was 92.231 nm, and the specific surface area associated with the BET technique was 1.2809 m^2^/g.

#### Scanning *electron* microscope (SEM)

**Figure 7:**
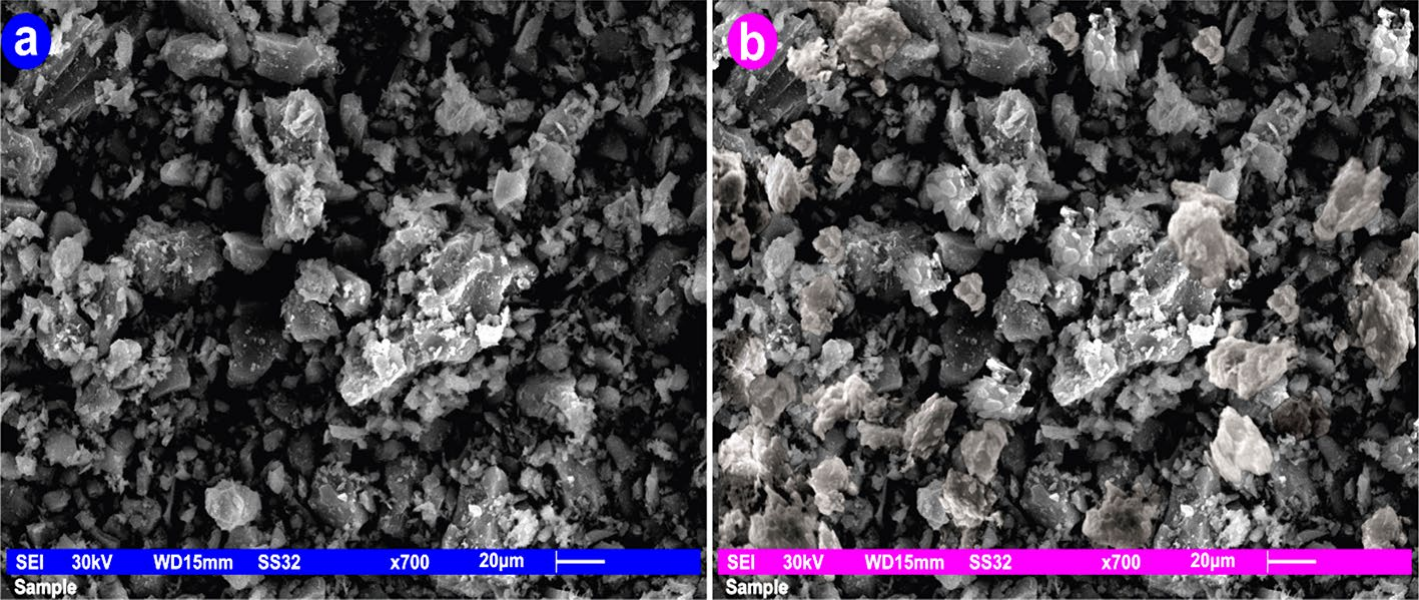
SEM of andesite at magnifications of 1 × 700 before and after Hg(II) adsorption.

**Figure 8:**
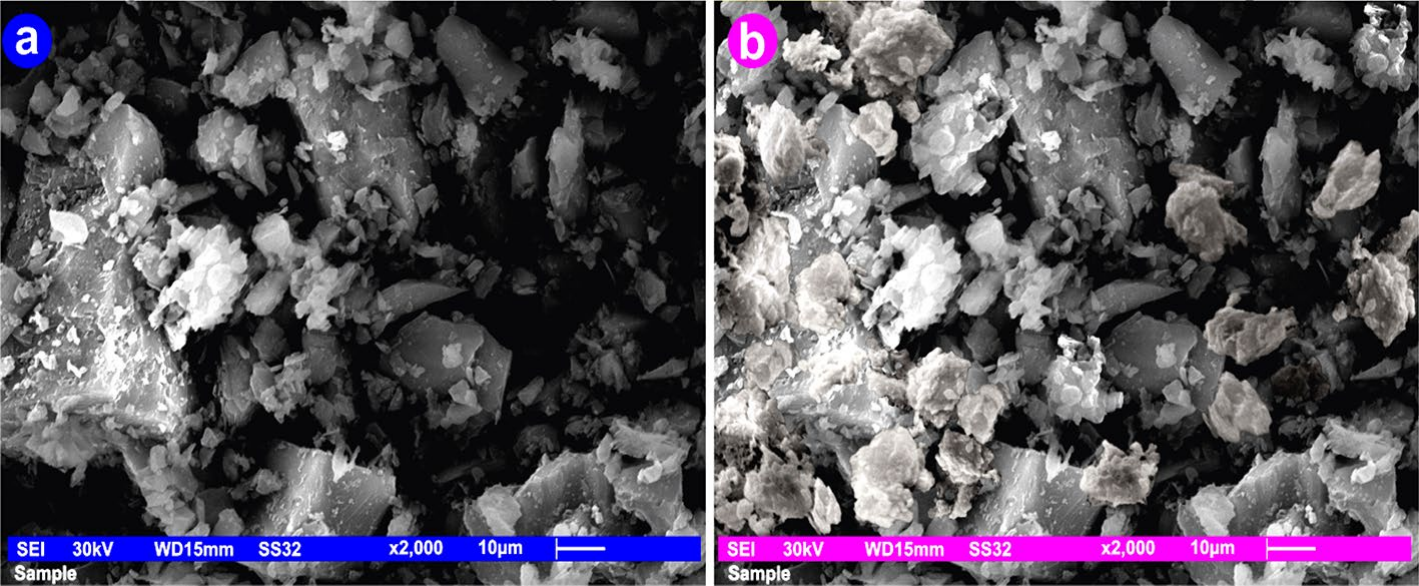
SEM of andesite at magnifications of 1 × 2000 before and after Hg(II) adsorption.

When andesite's porosity as an adsorbent was investigated using a scanning electron microscope (SEM, JEOL JSM 6510 Iv, England), it was discovered that andesite has both smooth and rough surfaces that resemble trenches with a number of lines that contain macrospores. The macrospores are clearly visible on the rough surfaces, and because of their presence, andesite functions well as a sorbent. The macrospores are what give the surface a high surface area. As can be seen in Figs. 7a and 8a, the adsorbent was found to be very porous at various magnifications, including 1 × 700 and 1 × 2000. The texture of the aluminium silicates was also discovered to be scattered with components such as Fe, Ca, Cu, K, and Na, among others.

Figures 7b and 8b demonstrate how Hg(II) was loaded onto the rough surface of andesite following adsorption.

#### Thermogravimetric analysis (TGA)

Using the TG-50 Shimatzu to analyze the thermal gravimetric characteristics of andesite over a temperature range of 22–800 °C, it was discovered that there was no change in weight or loss during the experiment, as shown in Fig. S1.

#### Transmission *electron* microscopy (TEM)

**Figure 9:**
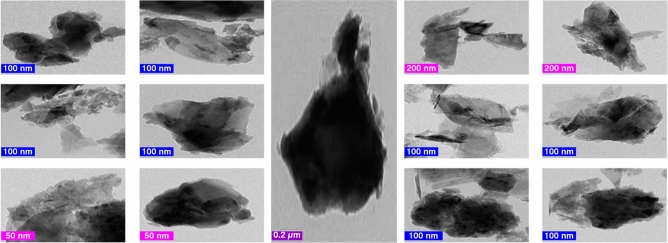
TEM analysis of andesite at different magnifications.

Figure 9 shows a rise in electron density before sorption in a TEM picture of andesite at different magnifications (5000–80,000). Heavy metals like iron can cause sorbent diffraction spots to appear. Their percentage on rough surfaces is far higher than that on smooth surfaces, and their number is clearly increasing.

#### Zeta potential

**Figure 10:**
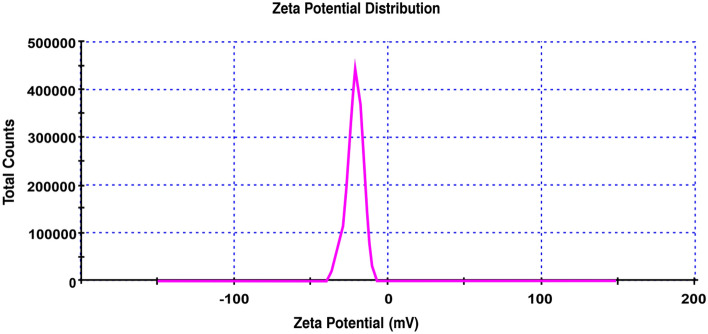
Zeta potential distribution of andesite.

Zeta potential size distribution value under these circumstances has been measured; cell description: clear disposable zeta cell; attenuator: 8; temperature (°C): 25; duration utilized (s): 60; count rate (kcps): 365.9; measurement location (mm): 5.50; Fig. S2 shows that the size average is 1033 d.nm. Additionally, under the same conditions as before (with the exception of the attenuator: 7), Fig. 10 shows that the zeta potential (mV) is − 22, indicating a negative charge and demonstrating the product's capacity to remove mercury (II) from aqueous solutions. This is because the pH value ≥ 7.50 is the isoelectric point at which the zeta potential is zero.

#### Equilibrium contact time influence

**Figure 11:**
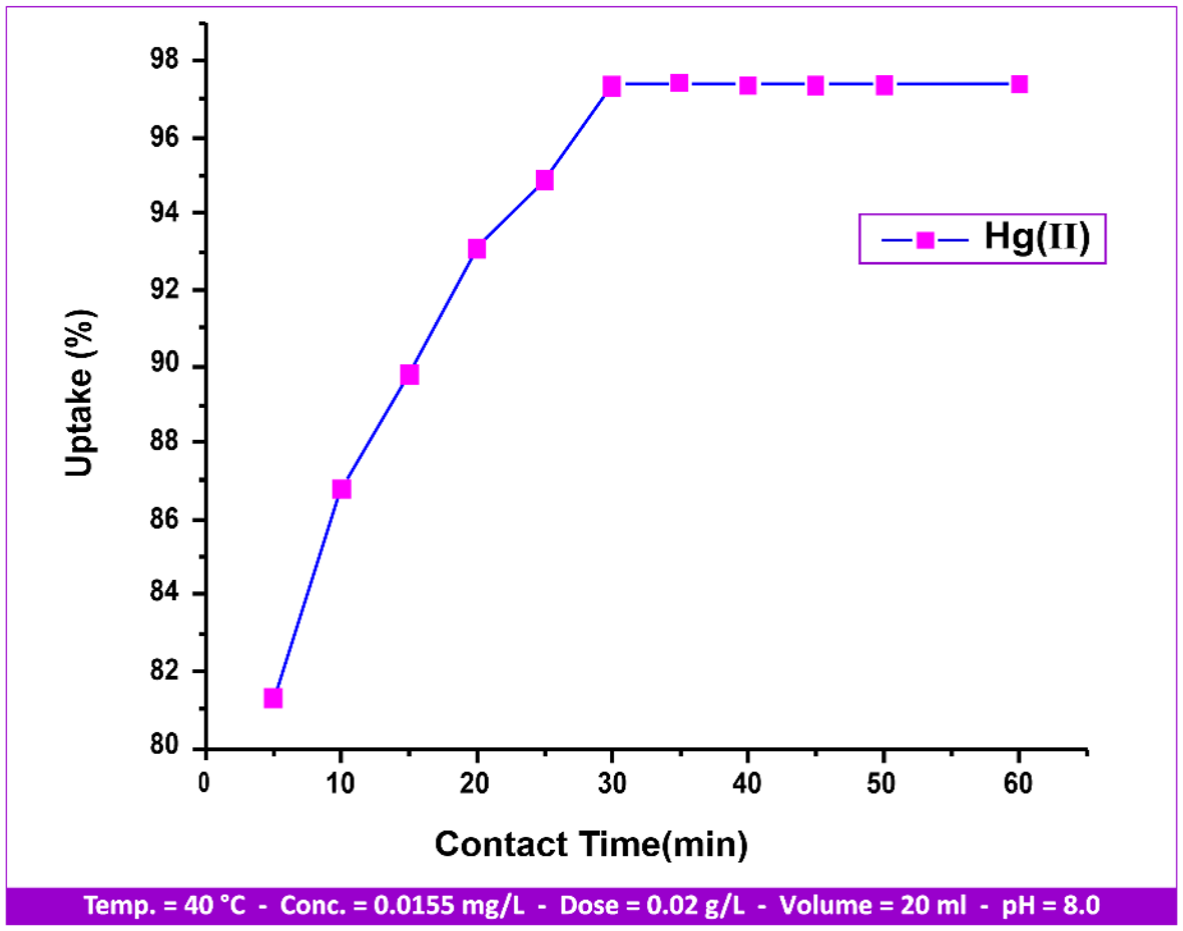
Contact time influence on Hg(II) uptake.

As seen in Fig. 11, the contact time of Hg(II) desorption was examined at different time intervals ranging from 5 to 60 min. There was a variance in the target adsorbate Hg(II) absorption percentage across all studies.

Following equilibrium, a centrifuge was used to separate the metal ion, and an inductively coupled plasma mass spectrometer^36,37^ was used to determine the filtrate. Using Fig. 11 as a guide, we observed that, up until equilibrium is attained at 30 min, the adsorption process may be exactly proportional to the contact time^38^. The attraction between the positive cation from the bulk liquid phase and the negative unoccupied adsorption sites^39^ aided in the adsorption process, as the adsorption uptake% and the number of active sites are closely connected. After 30 min, equilibrium was reached with an uptake percentage of 97.4%. After 30 min of shaking, the adsorption process reached equilibrium, and the andesite exhibited great affinity for Hg(II). From 5 to 30 min, uptake increases from 81.3 to 97.4%, and from 30 to 60%, uptake remains steady until saturation is reached.

#### Dose of adsorbent influence

**Figure 12:**
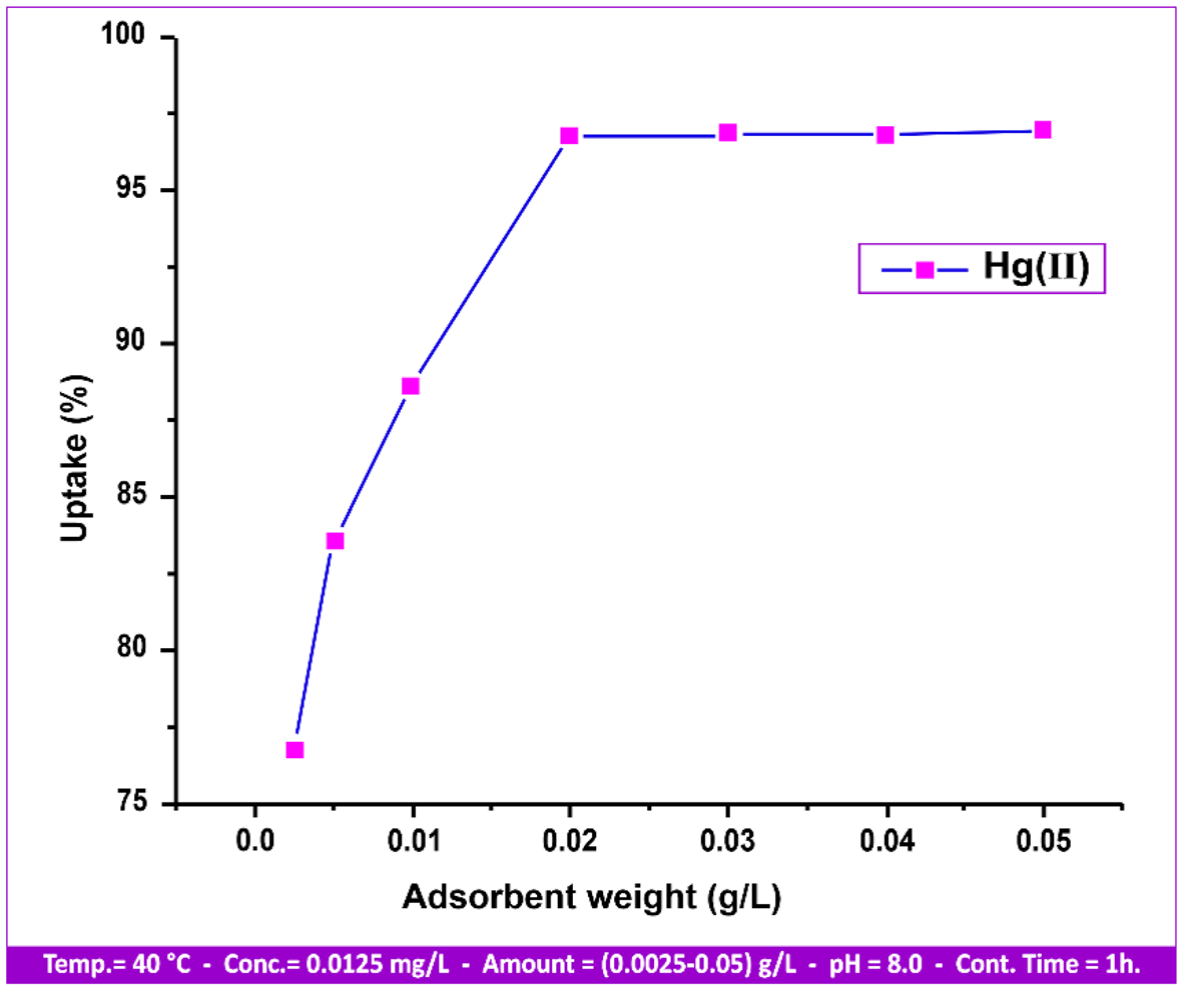
Various doses influence on Hg(II) uptake.

This study's main goal was to use the least quantity of adsorbent possible to remove the specified metal ion concentration. At 25 °C, pH 8.0, contact period of 30 min, stirring rate of 80 rpm, metal ion concentration of 0.0125 mg/l, and amount ranging from 0.0025 to 0.05 g/L, the impact of different doses was investigated (Fig. 12).

From 0.0025 to 0.05 g/L of sorbent, the adsorption (percentage of metal removed) increased and thereafter became nearly constant. Adsorption will continue until the sites are saturated because andesite has the ability to extract mercury by adsorption on the open active sites that are present on its surface. Subsequently, it was determined that a wide variety of possible adsorption sites existed on the clay surface, with a notable variation in energy based on the location of the site within the defect site^40^ or on the edge. It was subsequently determined that, depending on where the site was located on the edge or in the defect site, there were numerous types of possible adsorption sites on the clay surface with a considerable difference in energy. The nature of the adsorption sites was non-uniform and non-specific.

#### Effect of pH

**Figure 13:**
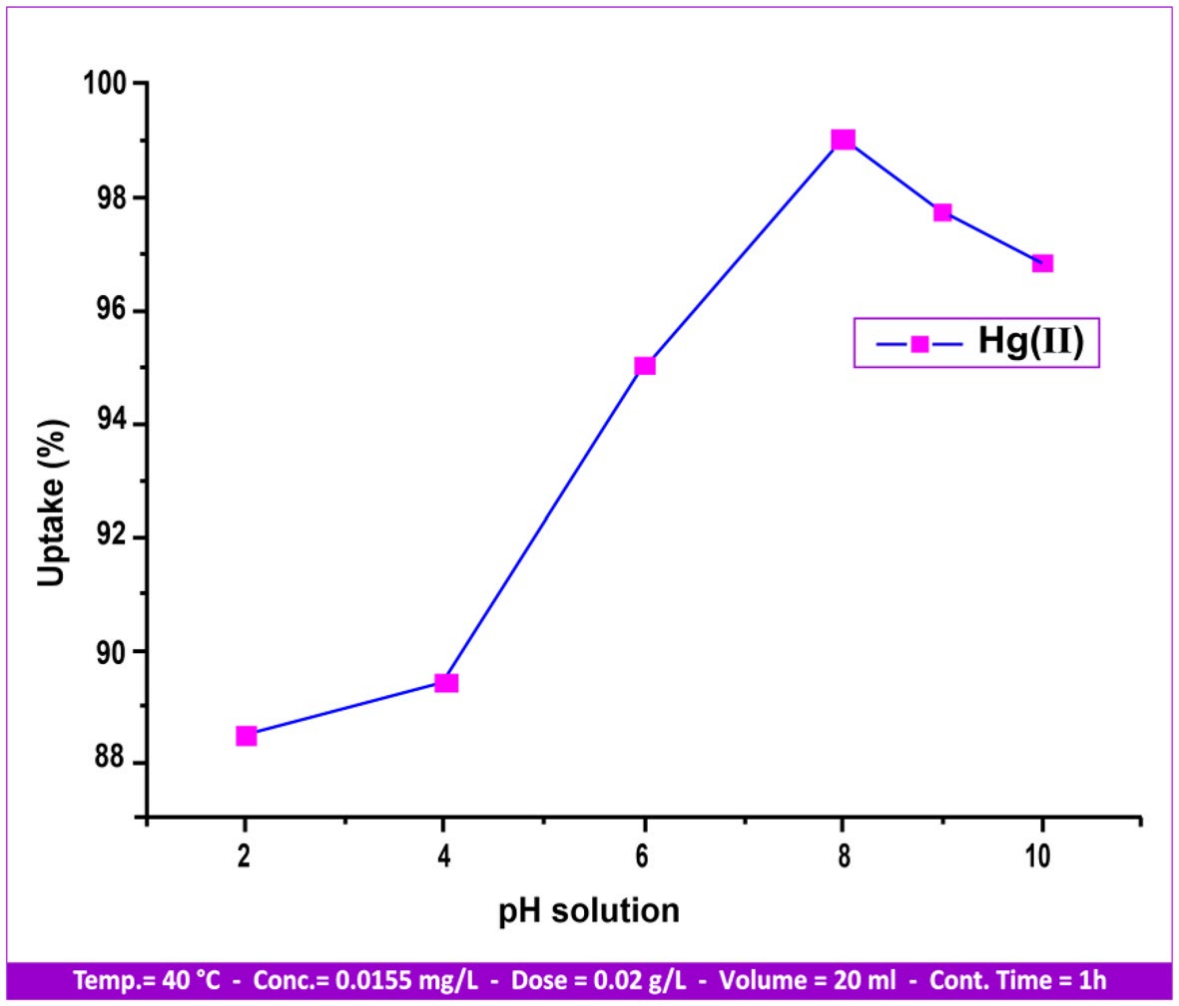
pH influence on Hg(II) uptake.

The study examined the impact of pH on adsorbent surface charge, ionization, and speciation of pollutants^41^ by varying the pH between 2 and 10 at 40 °C. The contact period was 30 min, with a stirring rate of 80 rpm and a metal ion concentration of 0.0155 mg/l. The effect of the initial solution pH on the adsorption is shown in Fig. 13, which shows that as pH increased, the absorption percentage increased in a direct proportion. The graph indicates that the adsorption rises from 2 to 8 and reaches its peak value at pH 8. The absorption percentage rose dramatically as the solution pH was raised from 2 to 10 [88.5%, 89.44%, 95.03%, 99.03%, 97.72%, and 96.84%]. This is explained by the negatively charged active sites on the andesite surface, such as aluminol (Al–OH) and silanol (Si–OH).

As a result, an electrostatic attraction developed between these sites and the positively charged ions (heavy metals)^42^. Thus, it is evident that at low pH values, the solution becomes acidic as the concentration of H^+^ increases, resulting in an increase in the competition between H^+^ and cations in the negative sites. Adsorption is then reduced, and conversely, at high pH values^43^, true sorption experiments are rendered unfeasible^44^ at pH values above 8.0 due to the precipitation of mercury metal hydroxide from solutions. Consequently, declines in the percentage removal were linked to both the ionized nature of the functional groups on the andesite surface and the generation of Hg(OH)_2_.

The solubility product constant (Ksp) is an equilibrium constant that sheds light on the balance between the solid solute and its dissociated constituent ions throughout the solution.

Solubility is dependent on several factors, the most significant ones being pH, the solvation enthalpy of the ions in the solution, and the lattice enthalpy of the salt.

Together with mercury, the anions chloride, hydroxide, and carbonate are all known to form effective complexes^45^. After pH 8.3, the concentrations of carbonate and hydroxide can affect the solubility by forming complexes, which cause the Ksp of mercury to drop and then tend to precipitate.

#### Metal ion concentration

Since the desired adsorbate concentrations were (0.0027, 0.0044, 0.0125, 0.0155, and 0.0399) mg/L, respectively, this variable was interpreted to provide an optimal concentration of Hg(II) that can be used to obtain a high uptake value on the andesite surface. According to the data shown in Fig. S3, at constant adsorbent concentrations, andesite has specific empty active sites that are attracted to metal ions up to the point of saturation. Therefore, even at constant concentrations, the number of ions sorbed on the surface increases as the concentration increases. This decrease in uptake was observed when the metal ion concentration was increased until stability was reached.

#### Temperature influences

**Figure 14:**
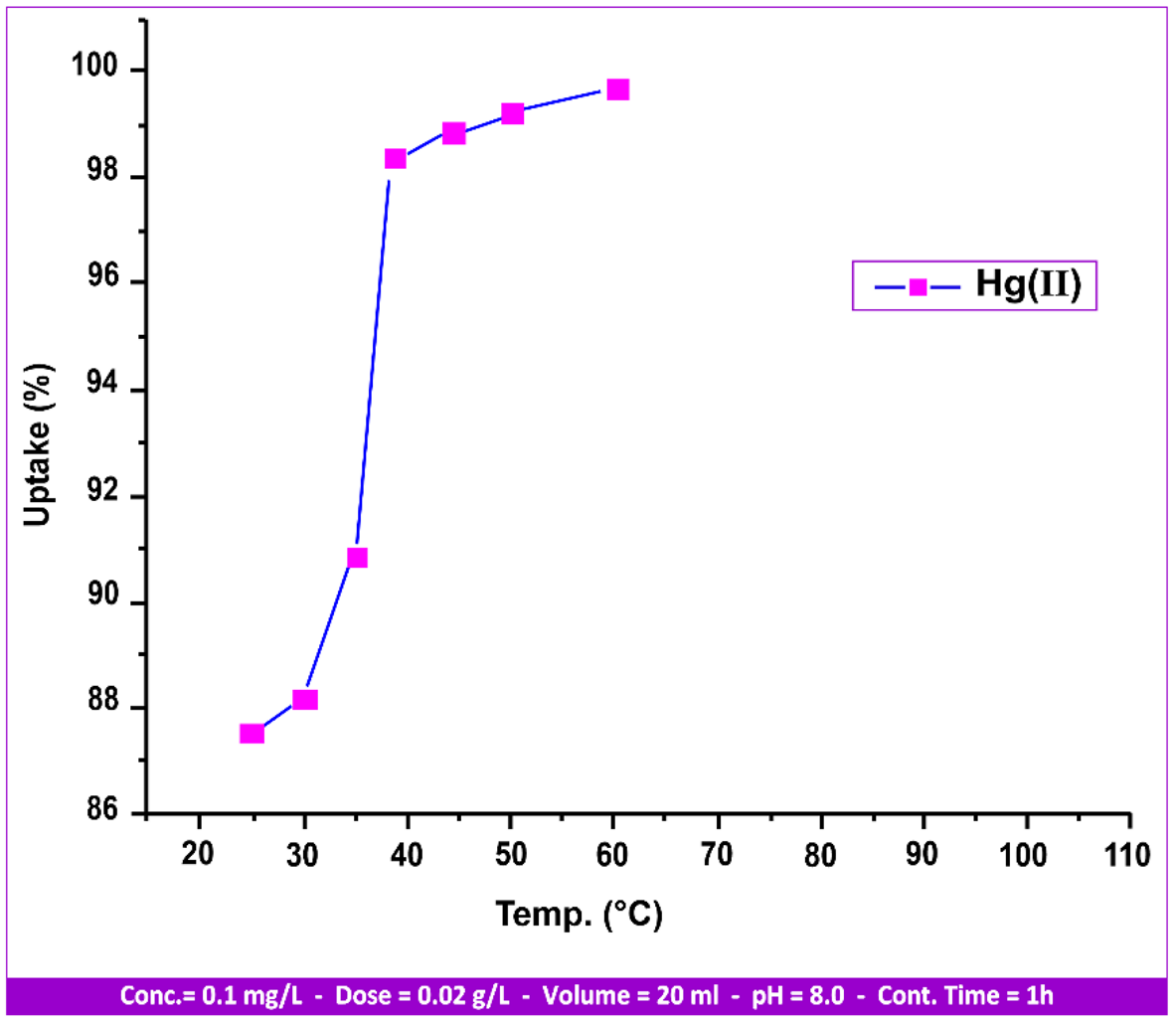
Temperature influences on Hg(II) uptake.

The research project investigated the connection involving temperature change and influence from 25 to 60 °C, as illustrated in Fig. 14. The temperature influence was measured at a 0.1 mg/L concentration at [T: (25, 30, 35, 40, 45, 50, 60) °C, pH: 8.00, stirring rate: 150 rpm, contact time: 1 h, sorbent dosage: 0.02 g/L]. Figure 14 shows the adsorption of Hg(II) on andesite with rising temperature, indicating that the reaction is endothermic. The solute's negative temperature coefficient of solubility^46^ could be the source of this reaction. In addition to an increase in the rate of sorbate^46^ diffusion among the particles and the desolvation of sorbing species, an increase in the ions' kinetics to be adsorbed may also be the cause of the rise in the uptake% of Hg(II) ions.

#### Adsorption isotherms

Isotherm equations, which deal with the physical adsorption of gases and vapors, provide the most important properties of industrial sorbents, such as pore volume, pore size or energy distribution, and specific surface area^47^. The simplest type of isotherm is the Langmuir isotherm. Since the Langmuir equation implies single-layer adsorption without molecular attraction on the surface of the adsorbate, it has limitations. However, by using this equation:

3 $${\text{C}}_{{\text{e}}} /{\text{q}}_{{\text{e}}} = {1}/\left( {{\text{q}}_{{\text{m}}} {\text{K}}_{{\text{L}}} } \right) + \left( {{1}/{\text{q}}_{{\text{m}}} } \right) \times {\text{C}}_{{\text{e}}}$$

The slope and interrupt of the Langmuir equation's linear figure plot can be used to specify the values of “q_m_” and “K_L_”.

One special Langmuir approach that is used to simulate multi-layer adsorption on heterogeneous surfaces is the Freundlich isotherm. It can be explained by the following equations^48^:

4 $${\text{q}}_{{\text{e}}} = {\text{K}}_{{\text{F}}} {\text{C}}_{{\text{e}}}^{\beta } = {\text{K}}_{{\text{F}}} {\text{C}}_{{\text{e}}}^{{{1}/{\text{n}}}}$$

“q_e_” is the equilibrium amount adsorbed per unit mass of adsorbent (mg/g); “C_e_” is the equilibrium concentration of the adsorbate in solution (mg/L); “K_F_” is the Freundlich isotherm constant (mg/g); and “n” is the adsorption intensity. The Freundlich equation´s logarithmic linear form is given as:

5 $${\text{q}}_{{\text{e}}} = {\text{log}}\left( {{\text{K}}_{{\text{F}}} } \right) + \left( {{1}/{\text{n}}} \right)\left( {{\text{log C}}_{{\text{e}}} } \right)$$

**Figure 15:**
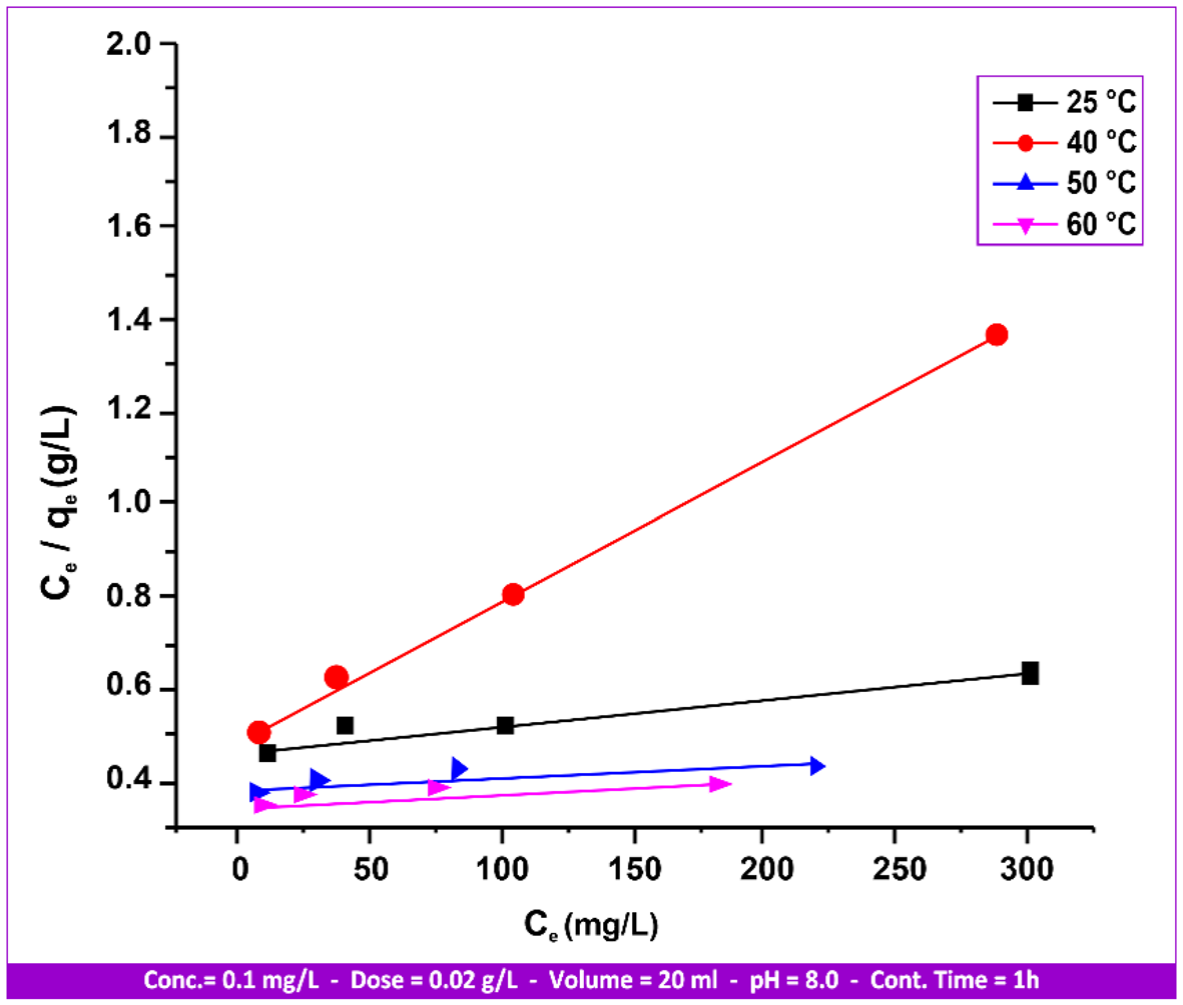
Hg(II) Langmuir isotherms on andesite range from 25 to 60 °C.

**Figure 16:**
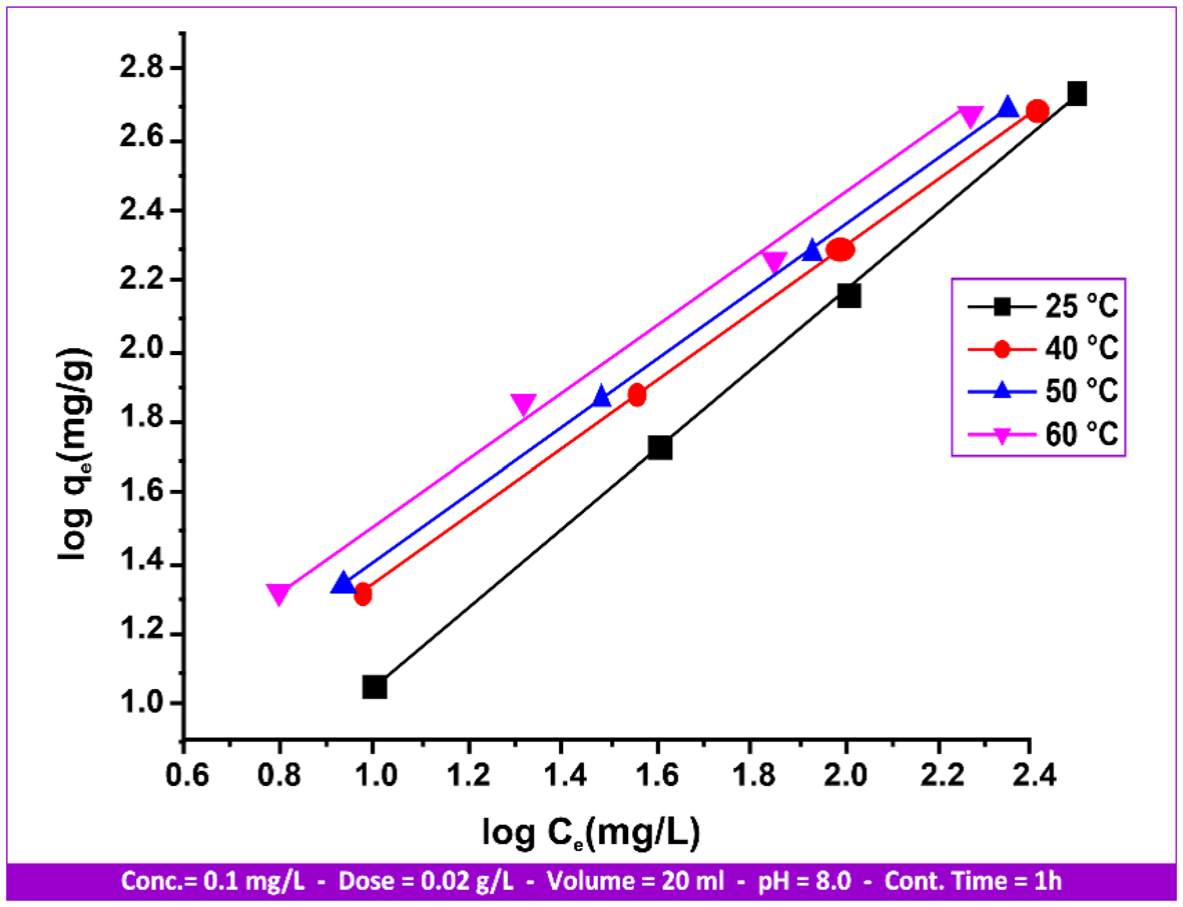
Hg(II) Freundlich isotherms on andesite range from 25 to 60 °C.

The mercury (II) adsorption on andesite was found to conform to the linear form of the Freundlich adsorption equation based on the relationship between all variables related to the adsorption isotherms (Langmuir and Freundlich) shown in Figs. 15 and 16, as well as with reference to the data mentioned in Table 4.

**Table 4:** Adsorption isotherm for Hg(II) andesite´s Langmuir and Freundlich parameters.

Temperature (°C)	Langmuir			Freundlich		
	K_L_ (L/mg)	R^2^	q _max_ (mg/g)	K_F_ (mg/g)	R^2^	n
25	1.125	0.8925	1.85	3.74	0.9990	1.10
40	0.978	0.9978	2.41	3.65	0.9993	1.06
50	0.11	0.8312	3.54	3.28	0.9997	1.85
60	1.50	0.9526	2.28	1.90	0.9942	1.10

Favorable adsorption is indicated by a value of n greater than 1, which means that as the solute concentration rises, so does the amount adsorbed. A value of n less than 1 denotes less favorable adsorption and is related to both a growing solute concentration and an increasing amount of solute adsorbed at a decreasing pace. Moreover, a value of n equal to 1 indicates a linear relationship between the amount adsorbed and the adsorbate concentration.

#### Kinetics of adsorption

There are two steps in the kinetic adsorption process. The adsorbate is assumed to have been transferred from the bulk solution to the adsorbent surface in the first stage. As a result, the adsorbent is distributed and organized inside the absorbent pores in the second step. The adsorption mechanism^49^ is also explained by the phase of figuring out the rate of the adsorption process. It puts out the theory that a first-order kinetic reaction is responsible for the adsorption rate constant.

6 $${\text{dq}}_{{\text{t}}} /{\text{d}}_{{\text{t}}} = {\text{k}}_{{1}} \left( {{\text{q}}_{{\text{e}}} - {\text{q}}_{{\text{t}}} } \right)$$

“k_1_” is the adsorption rate constant for first-order adsorption; “q_t_” is the amount of substrate adsorbed at time t (mg/g); and “q_e_” is the amount of substrate adsorbed at saturation (mg/g). The integration of Eq. (6) gives the following expression:

7 $${\text{Ln}}\left( {{\text{q}}_{{\text{e}}} - {\text{q}}_{{\text{t}}} } \right) = {\text{Ln}}\left( {{\text{q}}_{{\text{e}}} } \right) - {\text{k}}_{{1}} {\text{t}}$$

The linear plot of Ln(q_e_ − q_t_) versus t can yield “k_1_” and Ln(q_e_) values, respectively, from its slope and intercept. Adsorption is demonstrated to be a first-order kinetic reaction^49^ by comparing the “q_e_” values found at the plot intersections with experimental values.

**Figure 17:**
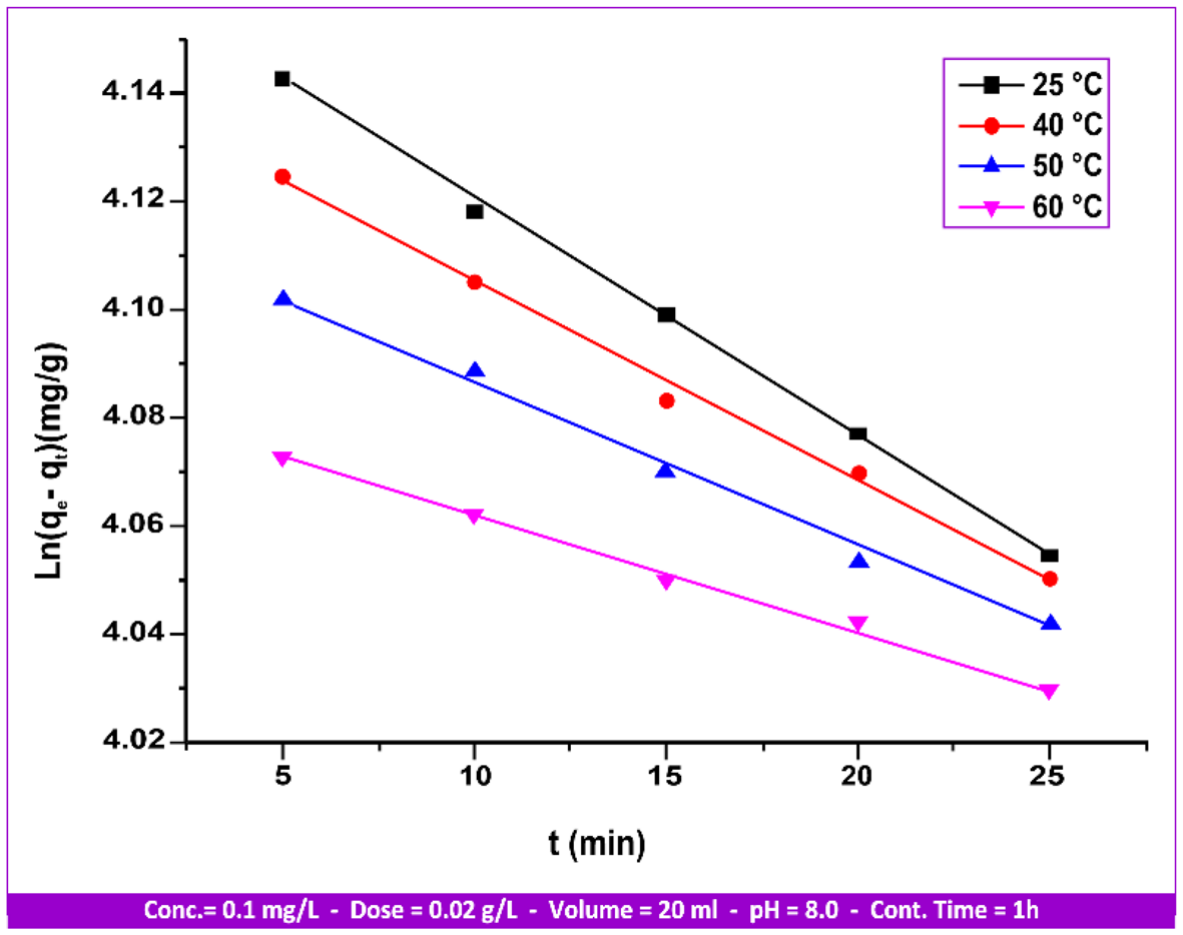
First-order kinetical curves of Hg(II) adsorption by andesite from 25 to 60 °C.

The adsorption kinetic curves of Hg(II) at 25, 40, 50, and 60 °C are readily visible in Fig. 17. In 30 min, the equilibrium time was attained. The correlation coefficient “R^2^” and the value of “k_1_” are computed and summarized in Table 5.

**Table 5:** Hg(II) adsorption first-order rate constant values on andesite as an adsorbent range from 25 to 60 °C.

Temperature (°C)	Hg(II)		
	q_e_ (mg/g)	k_1_ × 10^−3^ (g/mg/min)	R^2^
25	8.73	4.00	0.9966
40	9.93	3.00	0.9972
50	9.96	2.60	0.9977
60	9.96	2.00	0.9981

The expression obtained from applying the pseudo-second-order reaction kinetic^50^ is as follows:

8 $${\text{dq}}_{{\text{t}}} /{\text{d}}_{{\text{t}}} = {\text{k}}_{{2}} \left( {{\text{q}}_{{\text{e}}} - {\text{q}}_{{\text{t}}} } \right)^{{2}}$$

“k_2_” is the adsorption rate constant for second-order adsorption; “q_t_” is the amount of substrate adsorbed (mg/g) at time (t); and “q_e_” is the amount of substrate adsorbed at saturation (mg/g). The integration of Eq. (8) gives the following expression:

9 $${\text{t}}/{\text{q}}_{{\text{t}}} = {1}/{\text{k}}_{{2}} {\text{q}}_{{\text{e}}}^{{2}} + {\text{t}}/{\text{q}}_{{\text{e}}}$$

**Figure 18:**
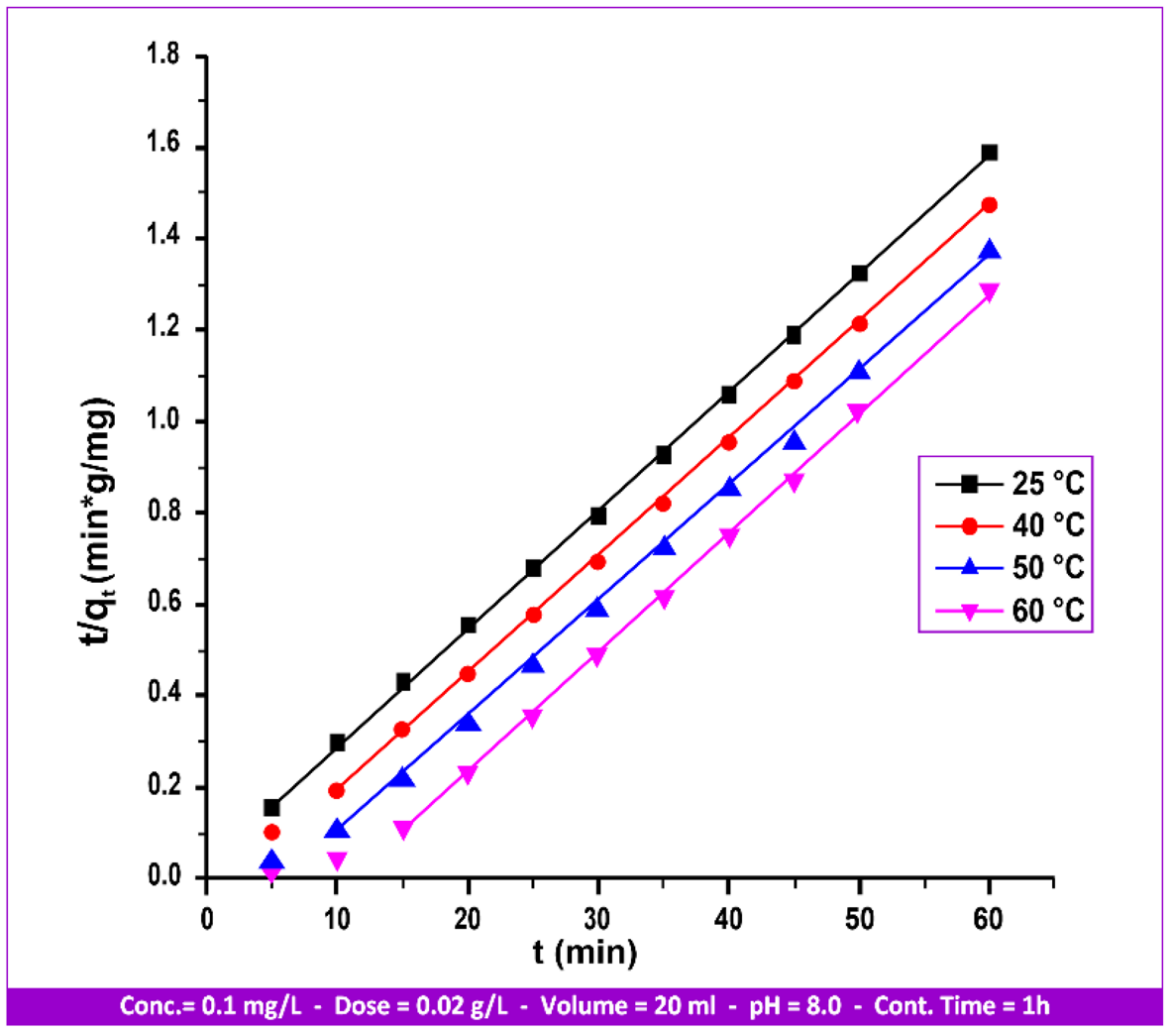
Second-order kinetical curves of Hg(II) adsorption by andesite from 25 to 60 °C.

A plot of t/q_t_ vs. t is used to derive the values of “q_e_” and “k_2_”_._ This provides a straight line as well as experimental and calculated values for “q_e_”. It was discovered that the adsorption kinetic curves of Hg(II) at 25, 40, 50, and 60 °C are displayed in Fig. 18.

Table 6 presents a summary of the correlation coefficient “R^2^” and the “k_2_” value. The Hg(II) sorption by andesite can be more accurately predicted by the pseudo-second-order kinetic model, as demonstrated by the preceding data presented in Tables 3 and 6 beside Figs. 17 and 18.

**Table 6:** Values of the metal ion Hg(II) adsorption second-order rate constant on andesite as an absorbent range from 25 to 60 °C.

Temperature (°C)	Hg(II)		
	q_e_ (mg/g)	K_2_ × 10^−3^ (g/mg/min)	R^2^
25	4.71	3.08	0.9996
40	4.84	3.24	0.9997
50	5.12	3.24	0.9997
60	7.95	13.00	0.9998

Each adsorption process's general methodology can be summed up in the following steps:

- The metal ions must come into contact with the adsorbent's boundary layer effect and diffuse onto the adsorbent surface from the boundary layer film.
- Diffusion of the metal ions into the adsorbent's porous structure, where intra-particle solute diffusion occurs in the adsorbed state or the particle's liquid-filled pores^35^.

**Figure 19:**
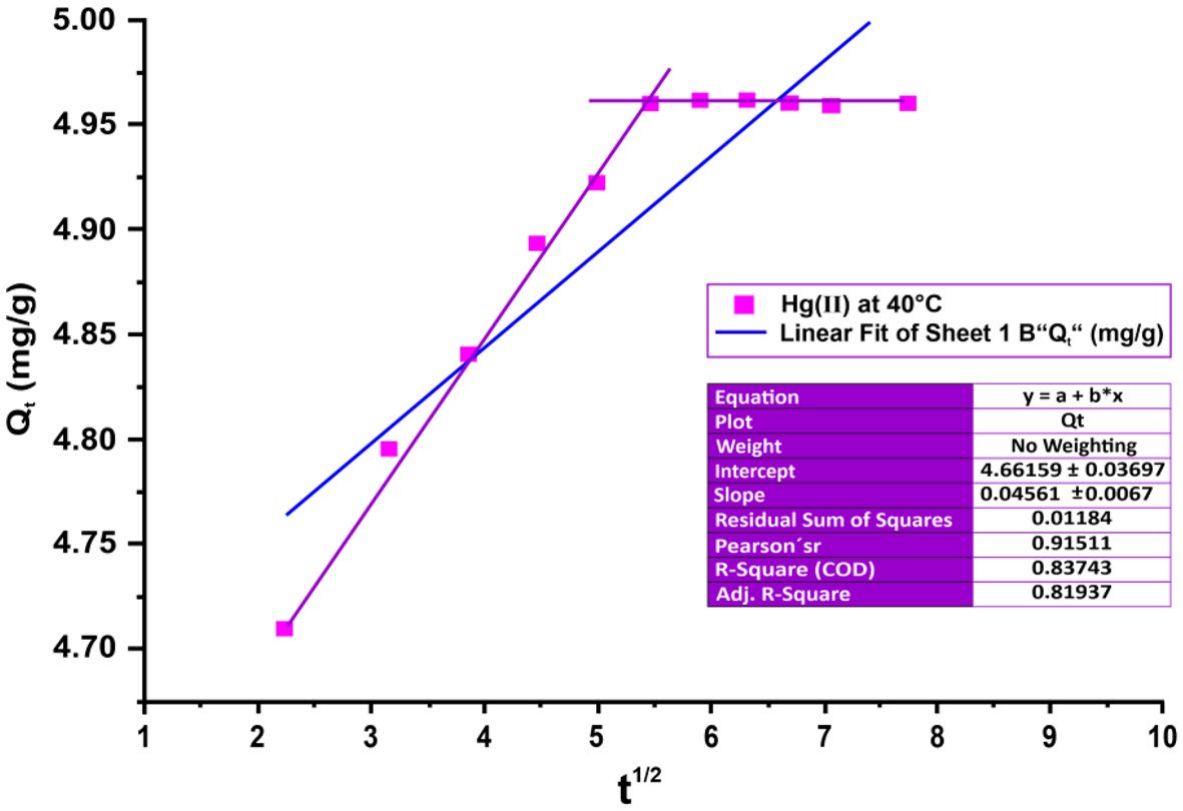
Intra-particle diffusion model for adsorption of Hg(II) on andesite as an absorbent at 40 °C.

The intra-particle diffusion model, proposed by Weber and Morris, is based on the equation:

10 $${\text{Q}}_{{\text{t}}} = {\text{K}}_{{{\text{pi}}}} {\text{t}}^{{{1}/{2}}} + {\text{C}}$$

where K_pi_ (mg/g/min^1/2^) is the intra-particle diffusion rate constant, and C (mg/g) describes the boundary layer thickness. The plot of Q_t_ vs. t^1/2^ in Fig. 19 and Table 7 reveals a linear relationship between slope (K_pi_) and an intercept (C).

**Table 7:** Values of the intra-particle diffusion model of metal ion Hg(II) adsorption on andesite as an absorbent at 40 °C.

Kinetic models	Hg(II)		
	K_pi_ (mg/g/min^1/2^)	C (mg/g)	R^2^
Intra-particle diffusion	0.045	4.661	0.837

The process methodology involves Hg(II) ions rapidly diffusing onto the surface of andesite through continuous stirring using a magnetic stirrer. Intra-particle diffusion of Hg (II) ions occurs in the porous structure of andesite, forming hydrogen bonds with hydroxyl groups on the porous structure until reaching equilibrium.

*Thermodynamic*

**Figure 20:**
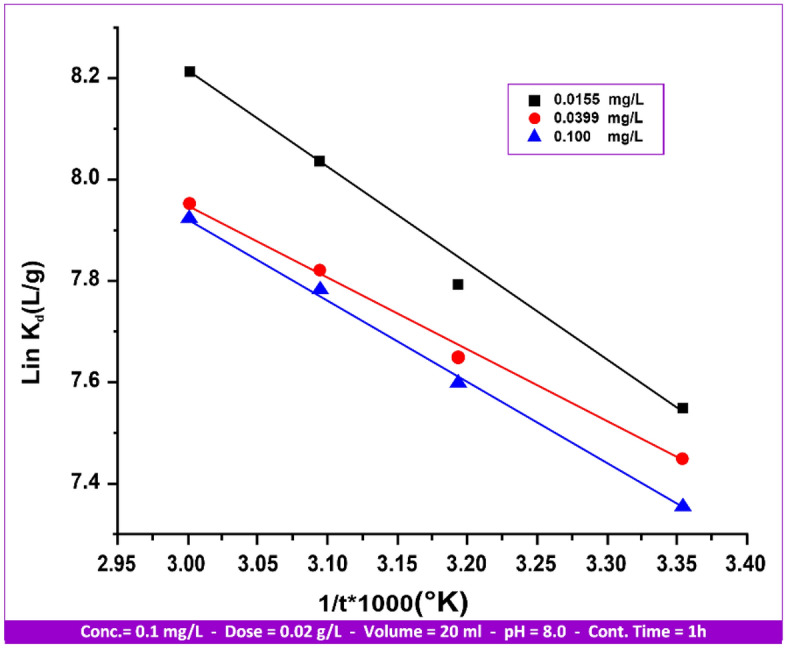
The impact of temperature on Hg(II) sorption on andesite´s thermodynamic behavior.

Using the following equation, the thermodynamic parameters were determined from the slope and intercept of ln K_d_ against 1/T (Fig. 20).

11 $${\text{ln K}}_{{\text{d}}} = (\Delta {\text{S}}/{\text{R}}){-}(\Delta {\text{H}}/{\text{RT}})$$

In this case, the distribution coefficient is denoted by “K_d_” and the entropy, temperature, gas constant, enthalpy, and “R” are all expressed in Kelvin. Using the well-known equation, the specific sorption's Gibbs free energy (ΔG) was determined.

12 $$\Delta {\text{G}} = \Delta {\text{H}}{-}{\text{T}} \cdot \Delta {\text{S}}$$

The values of the thermodynamic parameters for the sorption of Hg(II) on andesite are displayed in Table 8. A positive value of ΔH indicates an endothermic sorption process, as it is more advantageous at higher temperatures. It is noticed that the Gibbs free energy is negative and tends to gravitate towards the negative side as temperature rises. This implies that an induction phase is not necessary for the sorption process to occur naturally. Endothermic heat of adsorption may result from the extraction of water molecules from the sorbed cations and the solid/solution interface. The value of ΔG approaches the positive side as the concentration of Hg(II) ions rises. This indicates a reduction in spontaneity as the number of ions to be absorbed increases. Moreover, complication is promoted, and adsorption stability^46^ is indicated by a positive value of ΔS.

**Table 8:** Thermodynamic parameters for Hg(II) adsorption on andesite.

Hg(II) concentration (mg/L)	ΔH (kJ/mol)	ΔS (J/k/mol)	E_a_ (J/k/mol) (298–333)K	ΔG (kJ/mol)			
				298	313	323	333
0.0155	15.21	113.77	17.84	− 18.71	− 20.41	− 21.55	− 22.69
0.0399	8.44	91.12	11.07	− 18.72	− 20.09	− 21.00	− 21.91
0.1000	13.03	104.83	15.66	− 18.22	− 19.80	− 20.84	− 21.89

The energy of activation (E_a_) was determined by calculating the slope and intercept of the best-fit curve from the equation:

13 $$\Delta {\text{H}} = {\text{E}}_{{\text{a}}} - {\text{RT}}$$

*Adsorption mechanism*

**Figure 21:**
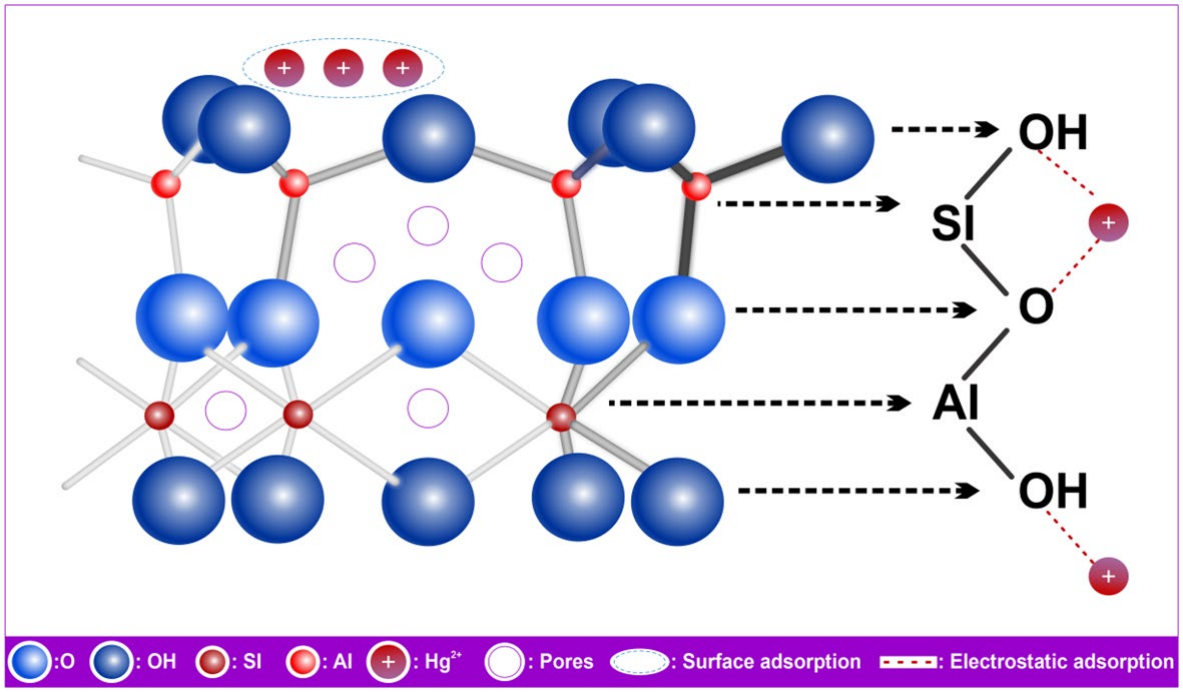
Adsorption mechanism of Hg(II) by andesite.

The mechanism has been demonstrated by the aforementioned investigation on andesite's adsorption of Hg(II) and its outcomes. Figure 21 illustrates the many adsorption mechanisms that could occur, such as electrostatics and surface sorption. Numerous pores with active sites that carry negative charges can be seen on the andesite surface. These sites, including aluminol (Al–OH) and silanol (Si–OH), are attracted to the positive ion Hg(II) by electrostatic forces, which greatly increase the surface adsorption capacity when H^+^ is lost from charged functional groups.

*Desorption study*

As desorption was nearly the opposite of the adsorption process, where the adsorption uptake% reached 99.03 and the metal ions were released from the andesite after 10 h, the results of the Hg(II) adsorption process showed that there is a major uptake percentage and high affinity between Hg(II) and andesite. It has also been evident that hydrochloric acid^38^, sodium chloride, and magnesium chloride were the most effective solutions when used to regenerate loaded kaolinite clays.

**Figure 22:**
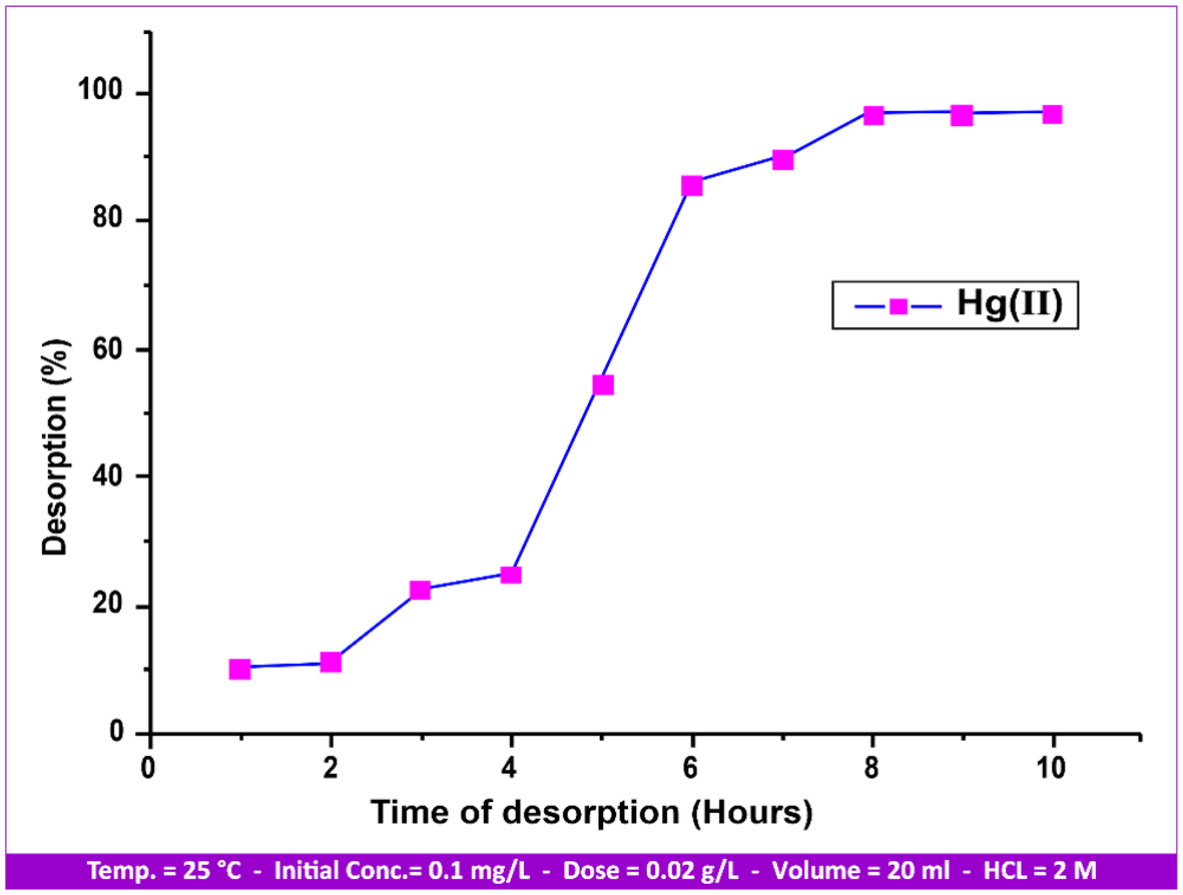
Desorption process of metal ion Hg(II) from andesite surface.

Due to its strong affinity for andesite, as can be observed in Fig. 22, it is evident that the Hg(II) desorption percent was initially lower. Another reason for this would be because specific sites absorbed more Hg(II) ions than generic sites did. Heavy metal ions Hg(II) that have been loaded on the adsorbent can be replaced by H^+^ ions during the regeneration process, which uses HCl (2 M) to form andesite, which may be used for further adsorption experiments. Figure S4 illustrates how the number of regeneration cycles causes the elimination rate of the heavy metal ion Hg(II) to decrease. The adsorption uptake percentage was 98.6% in the first cycle and reached 10% after 7 h.

*Application of andesite as an adsorbent*

Because of andesite's propensity to absorb some types of contaminants from wastewater, it has been used as an adsorbent. This issue could represent a serious challenge to the quality of the treated water that is used for other reasons^51^. Eight borosilicate bottles containing 200 ml of real samples were obtained from two tertiary wastewater treatment plants. Two of the bottles from plant 1, which has only one tank, were collected and acidified with 2% HNO_3_ to detect Hg^2+^, while the other sample was retained for the determination of NH_4_^+^, Cl^−^, Br^−^, NO_3_^−^, SO4^2−^, Na^+^, and K^+^. Using andesite under the following conditions: temperature of 25 °C, solution volume of 20 ml, contact time of 1 h, and adsorbent dose of 0.2 g/L, referring to Table 9**,** it was clear that andesite has a great ability to remove Hg^2+^, NH_4_^+^, and SO_4_^2−^ from wastewater samples, and on the other side, Cl^−^, Br^+^, K^+^, and NO_3_^−^ no influence, and Na^+^ has a slight effect**,** as the uptake% for removing Hg^2+^, NH_4_^+^, and SO_4_^2^ is 100, 99.2, and 100. For plant 2, six of the bottles were collected from tanks 1, 2, and 3, as each sample was used for Hg^2+^, H_2_S, and CH_3_SH analysis. All tests were carried out under the same circumstances as in plant 1; contaminants like mercury, hydrogen sulfide, and methanethiol were effectively eliminated. Results in Table 10 prove that andesite has excellent efficiency in removing Hg^2+^, H_2_S, and CH_3_SH from real wastewater, as the maximum uptake% was 97.53, 67.81, and 100, respectively, for the different three tanks related to plant 2.

**Table 9:** Real sample plant **1** during adsorption of NH_4_^+^, Cl^−^, Br^+^, NO_3**,**_ SO_4_^2−^, Na^+^, K^+^, and Hg^2+^ on andesite as an adsorbent at conditions (dose of adsorbent 0.2 g/L; temperature 25 °C; solution volume 20 ml; and contact time 1h).

Parameters	Before adsorption (mg/L)	After adsorption (mg/L)	Uptake%	Raw tank No
NH_4_^+^	0.10	0.0008	99.20	1
Cl^−^	184.00	183.00	0.54	1
Br^+^	8.90	8.90	0.00	1
NO_3_^−^	46.60	46.50	0.21	1
SO_4_^2−^	167.10	0.00	100.00	1
Na^+^	160.80	155.00	3.60	1
K^+^	30.10	30.00	0.33	1
Hg^2+^	0.002	0.00	100.00	1

**Table 10:** Real sample plant **2** during adsorption of H_2_S, CH_3_SH, and Hg^2+^ on andesite as an adsorbent at conditions (dose of adsorbent 0.2 g/L; temperature 25 °C; solution volume 20 ml; and contact time 1h).

Parameters	Before adsorption (mg/L)	After adsorption (mg/L)	Uptake%	Raw tank No
H_2_S	86.60	2.70	96.88	1
CH_3_SH	9.90	3.30	66.66	1
Hg^2+^	0.005	0.0001	98.00	1
H_2_S	99.90	3.90	96.09	2
CH_3_SH	8.70	2.80	67.81	2
Hg^2+^	0.002	0.00	100.00	2
H_2_S	89.00	2.20	97.53	3
CH_3_SH	7.80	2.90	62.82	3
Hg^2+^	0.005	0.0002	96.00	3

## Conclusions

Because andesite is a natural stone, it was supplied by Egypt. Since it was effectively used to adsorb Hg(II) at various concentrations from its aqueous solution, the experiments revealed an ideal contact time of 30 min, a pH of 8, a metal ion concentration of 0.0125 mg/L, a dose of 0.02 g/L, and a temperature of 40 °C. Conversely, the isotherm, kinetic, and thermodynamic studies employed optimal values. Ion exchange and the adsorption mechanism resulted in the Hg(II) adsorption by andesite. Andesite, with a specific surface area of 1.2809 m^2^/g and an average pore width of 92.231 nm, has a maximum adsorption capacity of 3.54 mg/g. The adsorption process is in accordance with the Freundlich isotherm model (R^2^ > 0.99) and pseudo-second order (R^2^ > 0.99). The process is endothermic, according to thermodynamic data, since ΔH and ΔS are both positive, while ΔG is negative, which indicates a spontaneous adsorption process. Based on an adsorption analysis, it is possible to achieve cost reduction through the reuse of andesite, which is essentially cost-effective. In the future, heavy metals from contaminated water will be extracted using andesite.

### Supplementary Information

Supplementary Figures.

## Data Availability

All data generated or analysed during this study are included in this published article [and its supplementary information files].
