# Supplementary material for: Adsorption characteristics and applications of andesite in removing some pollutants from wastewater
Source: Sci Rep. 2024 Jul 5;14:15523. doi: 10.1038/s41598-024-65043-y (PMC11226703; doi:10.1038/s41598-024-65043-y)
Supplement: Supplementary file 1 — Supplementary Figures. [file 41598_2024_65043_MOESM1_ESM.docx]

**
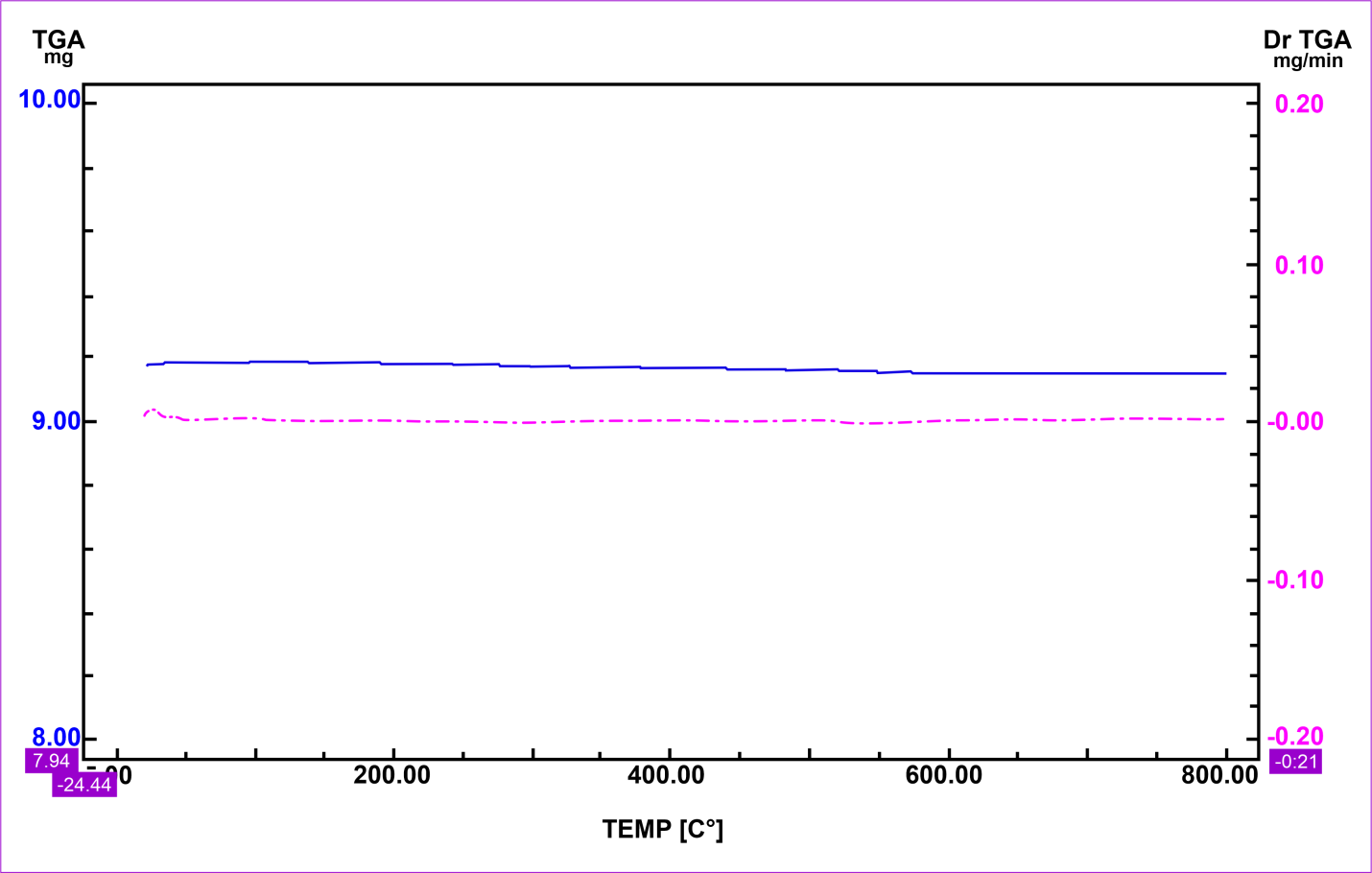
**

**Figure S1.** TGA analysis of andesite.


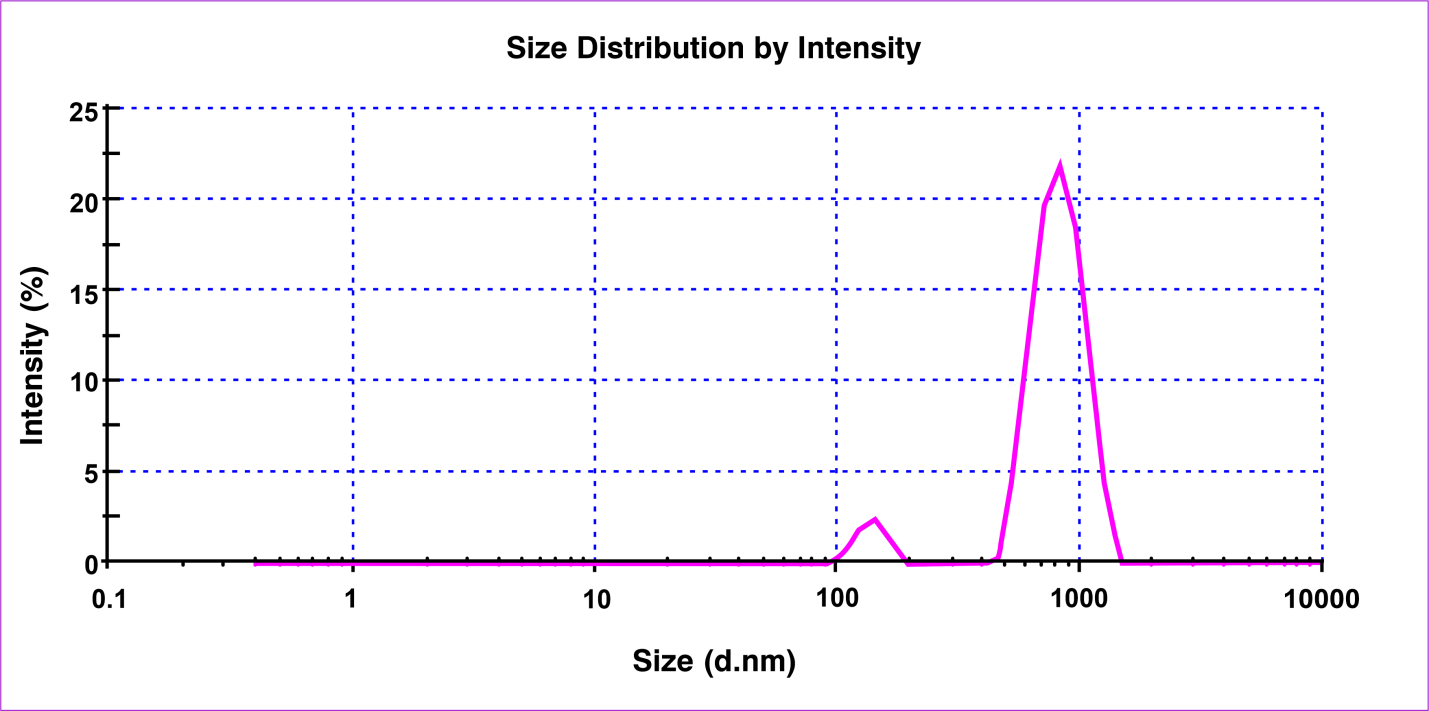


Figure S2. Number-wise distribution of sizes.

**
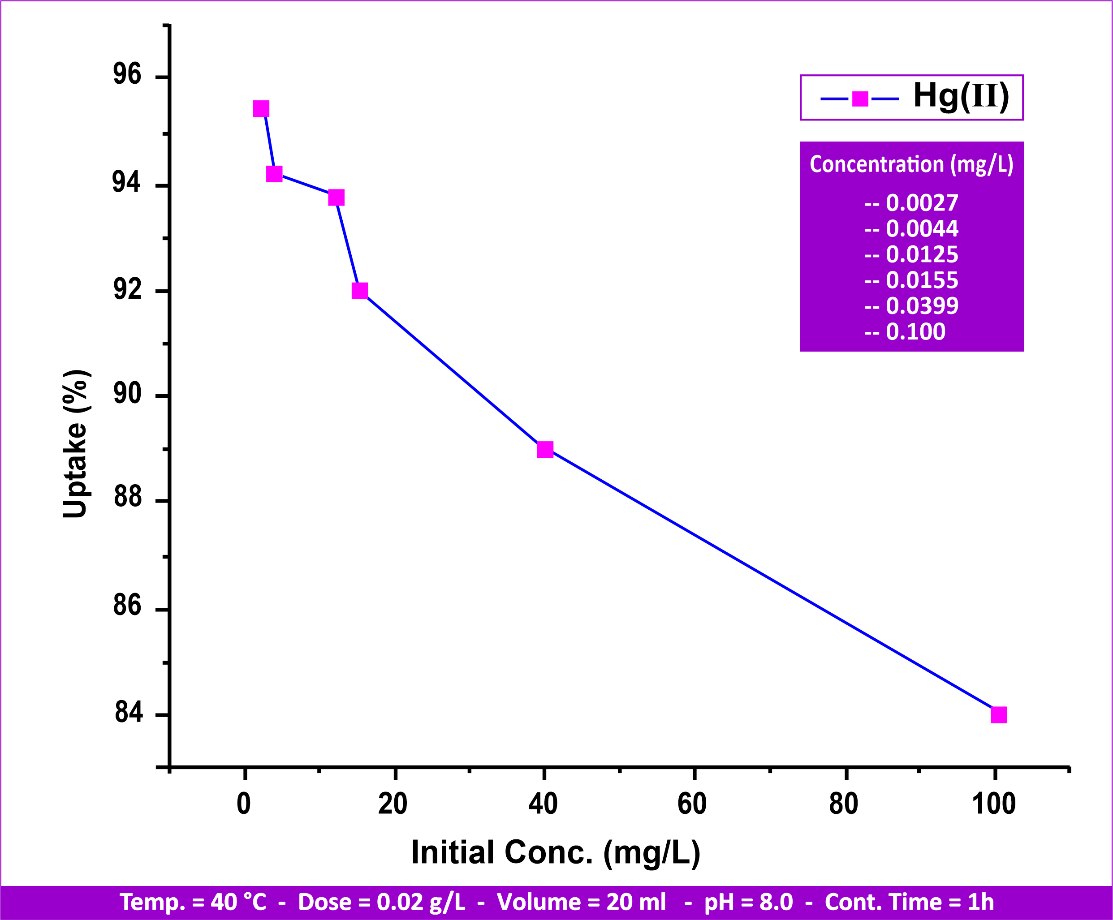
**

**Figure S3.** Metal ion concentration influence on Hg(II) uptake.

**
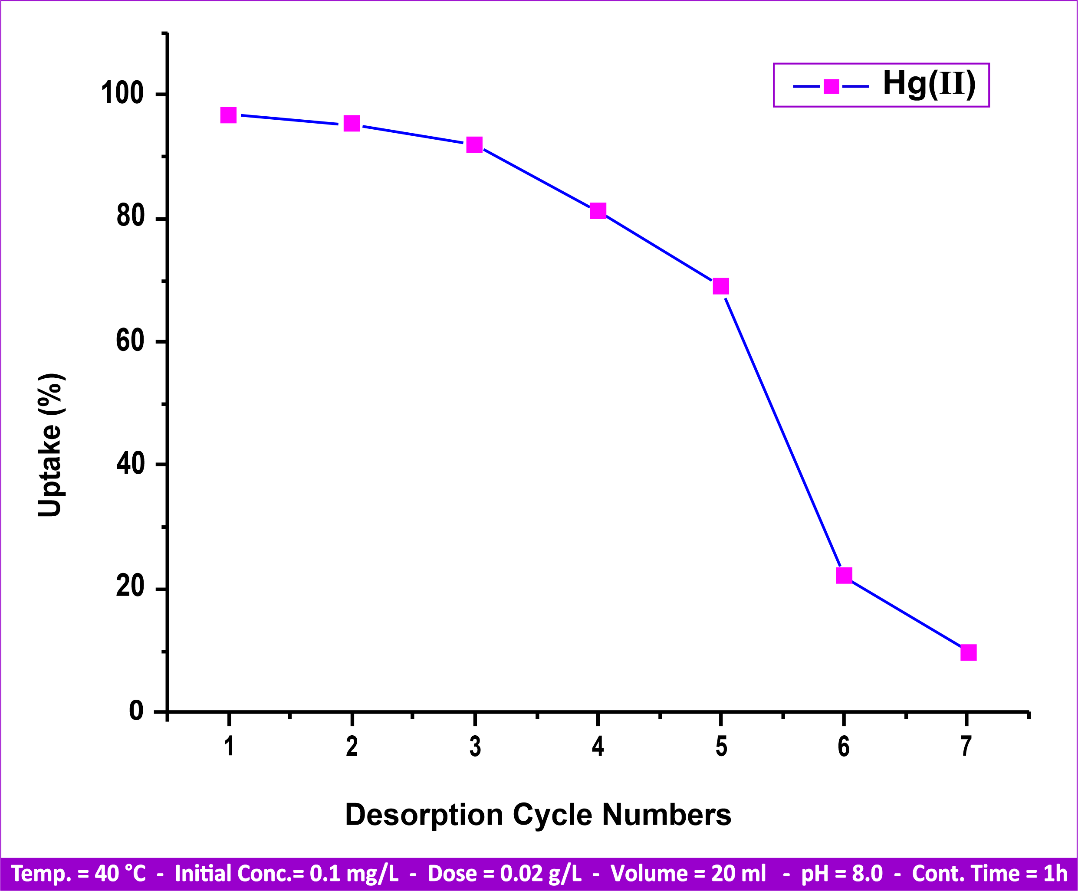
**

**Figure S4.** The influence of regeneration cycle numbers on the Hg(II) uptake.
